# Downregulation of Sirt6 by CD38 promotes cell senescence and aging

**DOI:** 10.18632/aging.204425

**Published:** 2022-12-06

**Authors:** Hongji Zhou, Shihai Liu, NanYang Zhang, Kehua Fang, Jinbao Zong, Yi An, Xiaotian Chang

**Affiliations:** 1 Medical Research Center, The Affiliated Hospital of Qingdao University, Qingdao, Shandong 266000, P.R. China; 2 Department of Cardiovascular Medicine, The Affiliated Hospital of Qingdao University, Qingdao, Shandong 266000, P.R. China; 3 Clinical Laboratory and Central Laboratory, The Affiliated Qingdao Hiser Hospital of Qingdao University, Qingdao, Shandong 266033, P.R. China

**Keywords:** CD38, cell senescence, aging, D-gal, Sirt6, C3G

## Abstract

Decreased nicotinamide adenine dinucleotide (NAD+) levels accompany aging. CD38 is the main cellular NADase. Cyanidin-3-O-glucoside (C3G), a natural inhibitor of CD38, is a well-known drug that extends the human lifespan. We investigated mechanisms of CD38 in cell senescence and C3G in antiaging. Myocardial H9c2 cells were induced to senescence with D-gal. CD38 siRNA, C3G and UBCS039 (a chemical activator of Sirt6) inhibited D-gal-induced senescence by reducing reactive oxygen species, hexokinase 2 and SA-*β*-galactosidase levels. These activators also stimulated cell proliferation and telomerase reverse transcriptase levels, while OSS-128167 (a chemical inhibitor of Sirt6) and Sirt6 siRNA exacerbated the senescent process. H9c2 cells that underwent D-gal-induced cell senescence increased CD38 expression and decreased Sirt6 expression; CD38 siRNA and C3G decreased CD38 expression and increased Sirt6 expression, respectively; and Sirt6 siRNA stimulated cell senescence in the presence of C3G and CD38 siRNA. In D-gal-induced acute aging mice, CD38 and Sirt6 exhibited increased and decreased expression, respectively, in myocardial tissues, and C3G treatment decreased CD38 expression and increased Sirt6 expression in the tissues. C3G also reduced IL-1*β*, IL-6, IL-17A, TNF-α levels and restored NAD+ and NK cell levels in the animals. We suggest that CD38 downregulates Sirt6 expression to promote cell senescence and C3G exerts an antiaging effect through CD38-Sirt6 signaling.

## INTRODUCTION

Aging is accompanied by a gradual decrease in nicotinamide adenine dinucleotide (NAD^+^) levels [1–5]. Treatments targeting NAD^+^ metabolism have emerged as a potential therapeutic strategy to ameliorate aging-related diseases and extend the human lifespan [6]. CD38, an ectoenzyme, is expressed on cell surfaces and catalyzes the production of cyclic adenosine diphosphate ribose (cADPR) from NAD^+^. CD38 has been identified as the main cellular NADase that downregulates NAD^+^ levels [7]. Thus, CD38 plays a central role in aging and the age-related decreases in NAD^+^ levels [8–10]. CD38 may serve as a pharmacological target to extend lifespan [11, 12]. However, the detailed mechanism by which CD38 modulates aging is not well understood.

In the present study, we cultured rat myocardial H9c2 cells with D-galactose (D-gal). D-gal has been verified to artificially induce senescence *in vitro* and *in vivo* [13]. Sirtuin 6 (Sirt6) is an NAD^+^-dependent protein deacylase and mono-ADP-ribosyltransferase of the sirtuin family. *In vitro* and *in vivo* studies have indicated that Sirt6 overexpression or activation exerts beneficial effects against aging [14]. We have detected high levels of CD38 expression in NK cells, in which CD38 suppresses Sirt6 expression to mediate immune cell function and cytokine secretion [15]. The present study used D-gal to treat myocardial H9c2 cells and determined the roles of CD38-Sirt6 axis in senescent progress. Cyanidin-3-O-glucoside (C3G), a family of flavonoid compounds, is a natural inhibitor of CD38. C3G has been confirmed to prevent CD38 from consuming NAD^+^ [16–18]. Many studies have reported that C3G exerts anti-tumor, anti-inflammatory and antioxidant effects [19–21]. C3G is well-known drug that extends the human lifespan [22, 23], but the functional mechanism has not been clearly elucidated. OSS-128167 is a selective inhibitor of Sirt6 [24, 25], and UBCS039 is an efficient Sirt6 activator [26, 27]. In the present study, D-gal-treated H9c2 cells were treated with a CD38-specific siRNA, Sirt6-specific siRNA, USBS039, OSS-128167 or C3G. Then, the expressions of cell senescence-associated genes, such as telomerase reverse transcriptase (TERT), SA-*β*-galactosidase (SA-*β*-gal) and hexokinase 2 (HK2), and the levels of reactive oxygen species (ROS) were examined to assess the essential role of the CD38-Sirt6 axis in cellular senescence. D-gal was injected into mice to produce symptoms of natural aging, and this method is often used as an animal model for acute aging [28]. In the present study also investigated CD38 and Sirt6 expression in myocardial tissues of the aging animals and observed the effects of D-gal, CD38-Sirt6 axis and C3G on the aging process.

## MATERIALS AND METHODS

### Cell culture

H9c2 cells were obtained from the Preservation of Typical Cultures of the Chinese Academy of Sciences. Cells were cultured in DMEM containing 10% FBS, 100 U/L penicillin and 100 U/L streptomycin at 37°C in a humidified incubator with 5% CO_2_. After 24 h of culture, adherent cells were treated with optimal concentrations of D-gal (Solarbio, China), C3G (Solarbio, China), OSS_128167 (Selleck, USA) or UBCS039 (Selleck) for 48 h. When H9c2 cells were treated with a combination of D-gal and C3G, the cells were incubated with D-gal at a final concentration of 10 g/L for 24 h, and then the culture medium was replaced with new culture medium containing D-gal at a final concentration of 10 g/L and C3G at a final concentration of 1 mM. The cells were continually cultured for 24 h. When H9c2 cells were treated with a combination of OSS_128167 and C3G, the cells were incubated with OSS_128167 at a final concentration of 100 μM for 24 h, and then the culture medium was replaced with new culture medium containing OSS_128167 at a final concentration of 100 μM and C3G at a final concentration of 1 mM. The cells were continually cultured for 24 h. When H9c2 cells were treated with a combination of D-gal and UBCS039, the cells were incubated with D-gal at a final concentration of 10 g/L for 24 h, and then the culture medium was replaced with new culture medium containing D-gal at a final concentration of 10 g/L and UBCS039 at a final concentration of 100 μM. The cells were continually cultured for 24 h.

### Cell viability analysis

The supernatant from the cultured H9c2 cells was discarded, and CCK-8 reagent (MCE, USA) was added to the culture. Cell proliferation was evaluated by measuring the spectrophotometric absorbance at 450 nm with Infinite 200 Pro multimode plate readers (TECAN, Switzerland).

### Cell apoptosis analysis

Cell apoptosis was measured using the Annexin V-FITC (fluorescein isothiocyanate)/PI Apoptosis (propidium iodide) Detection Kit (Elabscience, USA). H9c2 cells were collected by centrifugation and resuspended in Annexin V Binding Buffer from the kit. An Annexin V-FITC/PI apoptosis detection solution was added, and the stained cells were analyzed using a flow cytometer (Apogee A50, NovoCyte D2040R, UK).

### Real-time PCR

Total RNA was extracted from H9c2 cells using RNAiso Plus (Takara, Japan). The isolated RNA was reverse-transcribed into complementary deoxyribonucleic acid (cDNA) using HiScript III RT SuperMix (Vazyme, China). cDNA was added to ChamQ Universal SYBR qPCR Master Mix (Vazyme, China), and mRNA expression was examined by fluorescence-based real-time quantitative PCR using a LightCycle96 (Switzerland). Relative mRNA expression was analyzed using the 2−ΔΔCT calculation method. *β*-actin mRNA was used as an internal control to quantify the expression levels of target genes. The primer design is described in Supplementary Table 1.

### Detection of ROS in H9c2 cells

The intracellular ROS level was measured using the Reactive Oxygen Species Assay Kit (Beyotime Biotechnology, China). DCFA-DA solution from the kit was added to the treated H9c2 cells. The ROS level was measured using a fluorescence microplate reader (TECAN, Switzerland) with a 488-nm excitation wavelength and 525-nm emission wavelength.

### SA-β-galactosidase (SA-β-gal) cytochemical staining

SA-*β*-gal expression was measured using a senescence-associated *β*-galactosidase (SA-*β*-gal) staining kit (Beyotime, China). Cultured H9c2 cells were incubated with the fixative solution for 15 min. Following washing with PBS, the prepared dye in the kit was added, and the cells were incubated at 37°C overnight. The cells showing high SA-*β*-gal expression and activity were stained blue. A semi-quantitative analysis of SA-β-gal expression and activity was performed by counting the number of cells stained blue based on the positive cell counting function of ImageJ (Media Cybernetics, USA). The proportion of positive cells is the number of positive cells divided by the total number of cells per field.

### siRNA transfection

H9c2 cells were cultured for more than 24 h until 80% confluence. CD38 or Sirt6 siRNA was transfected into H9c2 cells using siRNA-Mate (Gene Pharma, China). Following 12 h of transfection, the cells were treated with D-gal, C3G, OSS_128167 or UBCS039 for 48 h. The Sirt6-specific siRNA target sequences were as follows: sense 5′-GUGCAUCUCAAUGGUUCCUTT-3′, and antisense 5′-AGGAACCAUUGAGAUGCACTT-3′. The CD38-specific siRNA target sequences were as follows: sense 5′-GCCUGAUCUAUACUCAAAUTT-3′ and antisense 5′-AUUUGAGUAUAGAUCAGGCTT-3′. Transfection of Allstars siRNA, which does not suppress the expression of any gene, was used as a control.

### Western blot analysis

H9c2 cells were cultured and collected by centrifugation. RIPA lysis buffer (Elabscience, China) was used to lyse the samples. Total protein was separated by electrophoresis with 12% sodium dodecyl sulfate–polyacrylamide gel electrophoresis (SDS–PAGE) and transferred to PVDF membranes. Anti-CD38 (ABclonal, China), anti-Sirt6 (CST, USA) and anti-*β*-actin antibodies (Elabscience, China) were incubated with the membranes. *β*-actin expression was used as an internal control. The relative expression levels of CD38 and Sirt6 were quantified using ImageJ software.

CD38 expression and Sirt6 expression in mouse myocardial tissues were detected by a similar protocol. The heart tissues were collected and lysed with RIPA lysis buffer (Elabscience, China) to extract total protein. A Pierce TM BCA protein assay kit (Thermo, USA) was used to quantify protein.

### Examination of CD38 expression in H9c2 cells using flow cytometry

The cultured H9c2 cells were collected after trypsin digestion and resuspended in PBS. The cells were incubated for 30 min at 4°C with allophycocyanin (APC)-conjugated anti-rat CD38 (BioLegend, USA) in the dark. The stained cells were analyzed using a flow cytometer.

### Animal aging model

Eight-week-old male C57BL/6J mice weighing 22 ± 2 g were purchased from Vital River Laboratory Animal Technology (Beijing, China). The mice were randomly divided into a normal control group (*n* = 20), a D-gal-induced aging group (*n* = 20), a C3G-treated group (*n* = 20) and a C3G and D-gal joint-treated group (*n* = 20). The normal control group was subcutaneously and daily injected with normal saline, the D-gal-induced aging group was subcutaneously and daily injected in the cervical dorsal region with D-gal (100 mg/kg) for 8 weeks, the C3G-treated group was subcutaneously and daily injected with normal saline for 8 weeks and then injected with a C3G solution (25 mg/kg) twice a week for 4 weeks through intraperitoneal injection, and the combination D-gal and C3G-treated group was injected daily with the D-gal solution (100 mg/kg) for 4 weeks and then injected with both D-gal (100 mg/kg) daily for 4 weeks and C3G (25 mg/kg) twice a week for 4 weeks.

The study was approved by the Ethics Committee of The Affiliated Hospital of Qingdao University (Approval number: QYFY WZLL 26622). Mouse care was carried out in accordance with the Helsinki Convention on Animal Protection.

### Hematoxylin and eosin (HE) staining

Mouse aorta samples were collected, fixed with 4% paraformaldehyde, embedded in paraffin and sectioned. Following deparaffinization and dehydration, the sections were stained with HE according to a routine protocol.

### Masson’s staining

Mouse aorta sections were incubated in a celestine blue solution. Following washing with water, the sections were incubated in fuchsine acid/Ponceau xylidine. After another wash, the sections were successively treated with a 1% phosphomolybdic acid solution, a 2% aniline blue acetate solution and 1% glacial acetic acid and then dehydrated with anhydrous ethanol before sealing and drying.

### Detection of lymphocyte subtypes in mouse peripheral blood

Peripheral blood from the experimental mice was collected in an EDTA blood collection tube and lysed with red blood cell lysis buffer (Solarbio, China). Leukocytes were centrifuged, and the cell pellet was resuspended in PBS. Cell subtypes were detected by flow cytometry. CD3+ CD19- T cells were detected with an APC-conjugated anti-mouse CD3e antibody (eBioscience, USA) and phycoerythrin (PE)-conjugated anti-mouse CD19 antibody (eBioscience, USA); CD3- CD19+ B cells were detected with APC-conjugated anti-mouse CD3e and PE-conjugated anti-mouse CD19 antibodies (eBioscience, USA); NK cells were detected with APC-conjugated anti-mouse CD3e antibody and PE-conjugated anti-mouse NK1.1 antibody (BioLegend, USA). These lymphocyte subtypes were detected with flow cytometry (ACEA BIO NovoCyte D2040R, USA). The proportion of each cell subtype was calculated as the proportion of that cell type in the total lymphocyte population.

### Detection of serum cytokines in mice

Peripheral blood was coagulated and centrifuged, and the supernatant was collected. The Mouse Inflammation Panel (13-plex) (BioLegend, USA) was used to detect the levels of cytokines in mouse blood. Mixed magnetic beads, assay buffer, a standard or test sample and the Mouse Inflammation Panel Detection solution including various anti-cytokine antibodies were mixed, and the mixture was incubated at room temperature in the dark for 3 h. Streptavidin-PE was added, and the incubation was continued for 0.5 h. The samples were centrifuged, and the pellet was resuspended in 1× wash buffer. The cytokine concentrations were measured by flow cytometry (Apogee A50, NovoCyte D2040R, UK).

### Evaluation of mouse metabolism

Mice were placed into a metabolic measurement cage for 24 h. Oxygen consumption (VO_2_) and carbon dioxide production (VCO_2_) were measured over 24 h using an Oxylet system (Panlab, Spain) with O_2_ and CO_2_ sensors coupled to a SEDACOM infrared system. The respiratory quotient (RQ) was calculated as the ratio of carbon dioxide production to oxygen consumption. Energy expenditure (EE) was calculated using the Weir equation (EE = 1.44 × VO_2_ × (3.815 + 1.23 × RQ)).

### Biochemical examination of mouse peripheral blood

Mouse peripheral blood was collected in a heparin blood collection tube. The peripheral blood was centrifuged, and the supernatant was collected. An automatic biochemical analyzer (Mindray, China) was used to detect the concentrations of alanine aminotransferase (ALT), aspartate aminotransferase (AST), creatine kinase (CK), lactate dehydrogenase (LDH), triglyceride (TG) and uric acid (UA) in the mouse plasma.

### Routine mouse blood test

Mouse peripheral blood was collected in an EDTA blood collection tube. An automatic hematology analyzer (Mindray) was used to detect the counts of WBCs, neutrophils (Neu), lymphocytes (Lym) and monocytes (Mon) in the blood.

### Immunohistochemistry

Paraffin sections of mouse heart tissues were incubated with an anti-CD38 polyclonal antibody (ABclonal, China) or anti-Sirt6 antibody (ZEN BIO, China) overnight at 4°C. The tissue sections were then treated with diaminobenzidine and stained with hematoxylin. The expression level was semi-quantified with ImageJ software (Media Cybernetics, USA). The system considers both staining intensity and stained area extent. The staining intensity and staining area of positive cells were calculated to determine the expression level of CD38 or Sirt6. The expression level was scored as follows: high positive = 3, positive = 2, low positive = 1 and negative = 0.

### Examination of NAD+ level and NAD+/NADH ratio in H9c2 cells

An NAD+/NADH assay kit (Beyotime Biotechnology, China) was used to measure NAD+ levels and the NAD+/NADH ratio in cultured H9c2 cells. Cells were collected and treated with lysis buffer. The cell lysates were treated with alcohol dehydrogenase at 37°C for 10 min in the dark. A chromogenic solution was added, and the mixture was incubated at 37°C for 30 min in the dark. NAD total (the sum of NAD+ and NADH) was measured at 450 nm with Infinite 200 Pro multimode plate readers (TECAN, Switzerland). To detect the level of NADH, cell lysates were incubated at 60°C for 30 min to decompose NAD+ and then treated with alcohol dehydrogenase at 37°C for 10 min. Following incubation at 37°C for 30 min in a chromogenic solution in the dark, the NADH level was measured at 450 nm. The NAD+ concentration and NAD+/NADH ratio were calculated as follows: NAD+ = [NAD total]-[NADH], and NAD+/NADH ratio = ([NAD total]-[NADH])/[NADH].

The myocardial tissues were washed with ice-prechilled PBS, placed in a homogenizer and mixed with NAD+/NADH extract solution (Beyotime Institute of Biotechnology, China) for homogenization on ice. The samples were subsequently centrifuged at 4°C, and the supernatant was collected for measurement.

### Statistical analysis

SPSS 24.0 software (Biomedical Computer Programs) was used to test the normality and homogeneity of variance. If it fits the homogeneity of variance and has a normal distribution, one-way ANOVA was used to test significance among multiple groups, and the LSD-T method was used for comparisons between two groups. If the data did not fit the normal distribution and homogeneity of variance, the Kruskal–Wallis test was used to test significance among multiple groups. Differences were considered statistically significant when the test standard *p* < 0.05.

## RESULTS

### Sirt6 is involved in D-gal-induced cell senescence

We treated H9C2 cells with different concentrations of D-gal to observe the inhibition of cell proliferation. D-gal at final concentrations of 0.1 g/L, 0.5 g/L, 1 g/L, 5 g/L and 10 g/L significantly reduced H9C2 cell proliferation, and D-gal at a final concentration of 10 g/L showed the most obvious inhibition of cell proliferation compared to untreated normal cells. C3G at final concentrations of 100 μM, 500 μM and 1 mM showed a significant stimulation on the cell proliferative activity, and 1 mM concentration of C3G exerted the strongest stimulation on the cell proliferation. Treatment with D-gal at a concentration of 10 g/L in combination with 0.1, 0.5 mM and 1 mM C3G significantly increased cell proliferation compared to H9C2 cells treated with D-gal at the same concentration. We then treated H9c2 cells with D-gal at a final concentration of 10 g/L and C3G at a final concentration of 1 mM in subsequent experiments. Similarly, we treated H9c2 cells with different concentrations of OSS-128167 to determine the most obvious inhibitory effect on cell proliferation and differential concentrations of UBCS039 to determine the most obvious effect to reverse the effect of D-gal on cell proliferation. OSS-128167 at final concentrations of 100 μM, 500 μM and 1 mM showed a significant inhibition of cell proliferative activity. We thus chose a concentration of 100 μM OSS-128167 for the next experiment. UBCS039 reversed the effect of D-gal at 100 μM and 200 μM on cell proliferation, and 100 μM showed a better effect than 200 μM. We thus chose a concentration of 100 μM UBCS039 for the next experiment (Supplementary Figure 1).

H9c2 cells were induced to undergo senescence by treatment with D-gal and were simultaneously treated with UBCS039, OSS-128167 or C3G. A cell proliferation assay was performed to observe the effects of these chemicals on cell viability. Cells treated with D-gal or OSS-128167 showed lower proliferation than untreated cells, while C3G treatment increased cell proliferation. Cells treated with C3G exhibited significantly higher proliferation than cells treated with OSS-128167 (Supplementary Figure 2A). Cells treated with UBCS039 or D-gal in combination with UBCS039 displayed significantly higher proliferation than cells treated with D-gal alone. Cells treated with D-gal in combination with UBCS039 showed a lower proliferation rate than cells treated with UBCS039 (Supplementary Figure 2B). The treated H9c2 cells were also examined by performing an apoptosis assay. Fewer cells treated with C3G and cells treated with UBCS039 underwent apoptosis compared with normal cells. Significantly fewer apoptotic C3G-treated cells were observed compared with the cells treated with OSS-128167 (Supplementary Figure 2C). A lower apoptosis rate was observed in cells treated with UBCS039 or D-gal combined with UBCS039 than in the cells treated with D-gal (Supplementary Figure 2D). Based on these results, D-gal and OSS-128167 decreased H9c2 cell viability, and C3G and UBCS039 increased cell viability.

**Figure 1:**
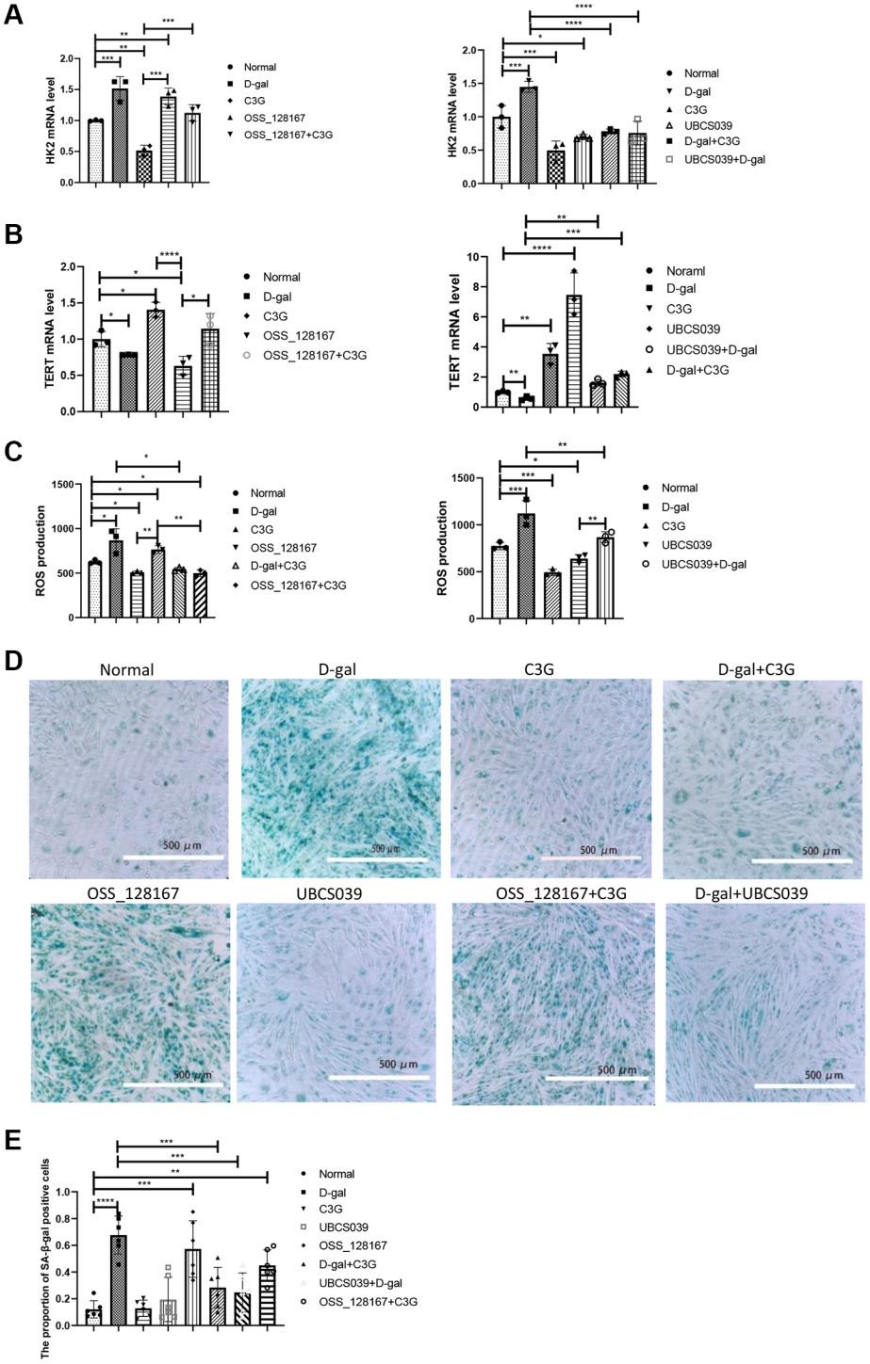
**D-gal and OSS-128167 increased ROS production and HK2 and β-gal expression and decreased TERT expression in H9c2 cells, whereas C3G and UBCS039 increased TERT expression and decreased ROS levels and HK2 and β-gal expression.** HK2 (**A**) and TERT mRNA levels in H9c2 cells (**B**) were detected using real-time PCR, ROS levels (**C**) were detected with a ROS assay, and *β*-gal expression (**D**) was observed using cytochemistry followed by semiquantitative analysis (**E**). Magnification, 10×. ^*^P < 0.05, ^**^P < 0.01, ^***^P < 0.001 and ^****^P < 0.0001.

The mRNA expression of senescence-related genes in H9c2 cells was measured using real-time PCR. Compared with normal cells, H9c2 cells treated with D-gal or OSS-128167 presented increased HK2 mRNA levels, while the HK2 mRNA level was decreased in C3G-treated cells and UBCS039-treated cells. HK2 mRNA levels in cells treated with C3G, UBCS039, D-gal combined with C3G or D-gal combined with UBCS039 were lower than those in the D-gal-treated cells. The HK2 mRNA level in C3G-treated cells was lower than that in OSS-128167-treated cells (Figure 1A). Additionally, the cells treated with D-gal or OSS-128167 exhibited lower TERT mRNA levels than normal cells, while the cells treated with C3G or UBCS039 showed increased TERT mRNA levels. TERT mRNA levels were higher in the cells treated with C3G, UBCS039, D-gal combined with C3G or UBCS039 combined with D-gal than in the D-gal-treated cells. TERT mRNA levels were also higher in cells treated with C3G or both OSS-128167 and C3G than in OSS-128167-treated cells. TERT mRNA levels were lower in the cells treated with UBCS039 combined with D-gal than in the cells treated with UBCS039 (Figure 1B).

The ROS level was measured in the treated H9c2 cells. The OSS-128167 or D-gal combined with OSS-128167 showed higher ROS levels than that in the cells treated with D-gal, while the cells treated with C3G showed lower ROS levels. The cells treated with C3G or OSS-128167 combined with C3G showed lower ROS levels than OSS-128167-treated cells. ROS levels were significantly lower in the cells treated with C3G or D-gal combined with C3G than in the cells treated with D-gal. ROS levels were significantly lower in the cells treated with UBCS039 or D-gal combined with UBCS039 than in the cells treated with D-gal. ROS levels in normal cells and cells treated with D-gal combined with UBCS039 were significantly higher than those in UBCS039-treated cells (Figure 1C).

SA-*β*-gal expression in treated H9c2 cells was stained with a kit. Semi-quantitative analysis showed that the proportion of SA-*β*-gal-expressing cells was higher in cells treated with D-gal or OSS-128167 than in normal cells. The proportion of positive cells was lower in the cells treated with C3G, UBCS039, D-gal combined with C3G or D-gal combined with UBCS039 than in the cells treated with D-gal alone (Figure 1D and 1E). Thus, D-gal induces cellular senescence, and the activities of C3G and Sirt6 exert an antiaging effect; furthermore, Sirt6 is involved in this process.

### CD38 exhibits increased expression to downregulate Sirt6 expression in cellular senescence

**Figure 2:**
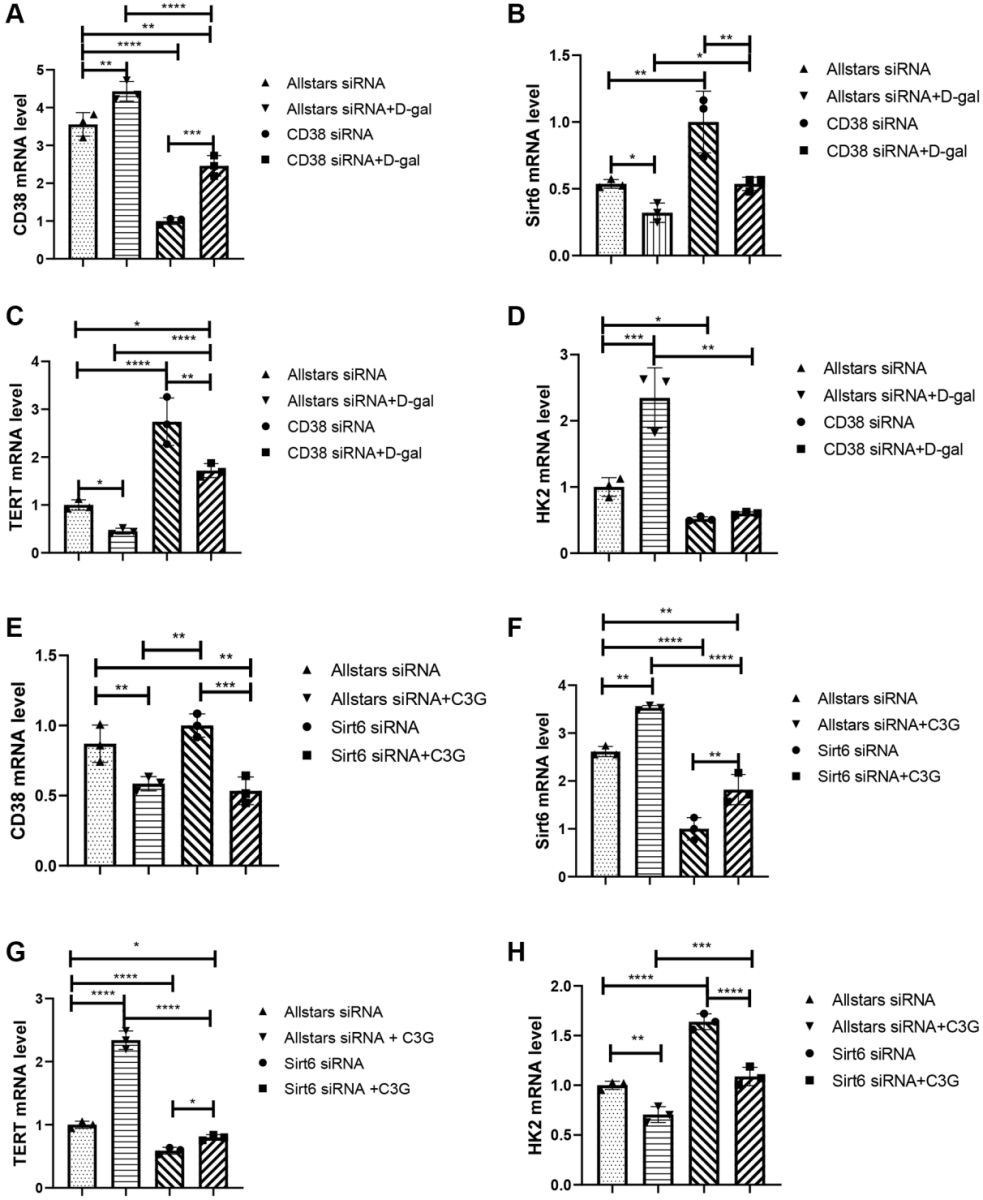
**Treatment with CD38 siRNA and C3G decreased HK2 mRNA expression and increased Sirt6 and TERT expression in H9c2 cells, and Sirt6 siRNA and D-gal treatment increased HK2 expression and deceased TERT expression.** Real-time PCR was used to detect the CD38 mRNA level (**A**), Sirt6 mRNA level (**B**), TERT mRNA level (**C**), and HK2 mRNA level (**D**) in cells after CD38 siRNA transfection. Real-time PCR was also performed to detect the CD38 mRNA level (**E**), Sirt6 mRNA level (**F**), TERT mRNA level (**G**), and HK2 mRNA level (**H**) in cells after Sirt6 siRNA transfection. ^*^P < 0.05, ^**^P < 0.01, ^***^P < 0.001 and ^****^P < 0.0001.

H9c2 cells were transfected with a CD38-specific or Sirt6-specific siRNA and then treated with D-gal. Transfection by CD38 siRNA led to significantly lower CD38 mRNA expression and higher Sirt6 mRNA expression than that of transfection by Allstars siRNA in H9c2 cells, while D-gal-treated cells transfected by Allstars siRNA showed increased CD38 mRNA expression and decreased Sirt6 mRNA expression. The cells transfected with CD38 siRNA followed by D-gal treatment or Allstars siRNA followed by D-gal treatment resulted in significantly higher CD38 mRNA levels and lower Sirt6 mRNA levels than that of the cells transfected with CD38 siRNA. Cells transfected with CD38 siRNA followed by D-gal treatment showed lower CD38 mRNA expression and higher Sirt6 mRNA expression than that of cells transfected with Allstars siRNA followed by D-gal treatment. The results indicated that CD38 exhibited increased expression, which decreased Sirt6 expression in D-gal-induced senescent cells (Figure 2A and 2B). TERT mRNA levels in cells transfected with CD38 siRNA or CD38 siRNA followed by D-gal treatment were higher, while the cells transfected with Allstars siRNA followed by D-gal treatment showed a lower mRNA level than that in the cells transfected with Allstars siRNA alone. TERT mRNA levels in the cells transfected with Allstars siRNA followed by D-gal treatment or CD38 siRNA followed by D-gal treatment were lower than those in cells transfected with CD38 siRNA alone. TERT mRNA levels were higher in the cells transfected with CD38 siRNA followed by D-gal treatment than in cells transfected with Allstars siRNA followed by D-gal treatment (Figure 2C). Cells transfected with Allstars siRNA followed by D-gal treatment exhibited higher HK2 mRNA expression levels than H9c2 cells transfected with Allstars siRNA. Cells transfected with CD38 siRNA followed by D-gal treatment showed lower HK2 mRNA expression than cells transfected with Allstars siRNA followed by D-gal treatment (Figure 2D). CD38 mRNA levels were significantly lower in cells transfected with Allstars siRNA followed by C3G treatment than in cells transfected with Allstars siRNA, and Sirt6 mRNA expression was significantly increased, while the levels in cells transfected with Sirt6 siRNA were significantly decreased. CD38 mRNA levels were lower in cells transfected with Sirt6 siRNA followed by C3G treatment than in cells transfected with Sirt6 siRNA. Sirt6 mRNA expression in cells transfected with Allstars siRNA, transfected with Allstars siRNA followed by C3G treatment, or Sirt6 siRNA followed by C3G treatment was higher than that in cells transfected with Sirt6 siRNA alone. The cells transfected with Sirt6 siRNA followed by C3G treatment showed lower Sirt6 mRNA expression than cells transfected with Allstars siRNA followed by C3G treatment. The results indicated that C3G inhibited CD38 expression and elevated Sirt6 expression (Figure 2E and 2F). TERT mRNA expression was higher in cells transfected with Allstars siRNA, Allstars siRNA followed by C3G treatment, or Sirt6 siRNA followed by C3G treatment than in cells transfected with Sirt6 siRNA. C3G-treated cells showed lower TERT mRNA expression after Sirt6 siRNA transfection than cells treated with C3G after Allstars siRNA transfection. The results indicated that C3G and Sirt6 suppressed cell senescence and that Sirt6 plays a role under C3G regulation (Figure 2G). HK2 mRNA expression was lower in cells transfected with Allstars siRNA followed by C3G treatment than in cells transfected with Allstars siRNA, while the expression was increased in Sirt6 siRNA-transfected cells. HK2 mRNA was lower in cells transfected with Sirt6 siRNA followed by C3G treatment than in cells transfected with Sirt6 siRNA alone. Cells transfected with Sirt6 siRNA followed by C3G treatment showed higher HK2 mRNA expression than cells transfected with Allstars siRNA followed by C3G treatment. The results indicated that C3G and Sirt6 suppressed HK2 expression and that Sirt6 plays a role under C3G regulation (Figure 2H). The above result indicated that C3G decreases CD38 expression and increases Sirt6 expression, and C3G suppresses cellular senescence by increasing Sirt6 expression. Sirt6 expression also inhibits cellular senescence.

**Figure 3:**
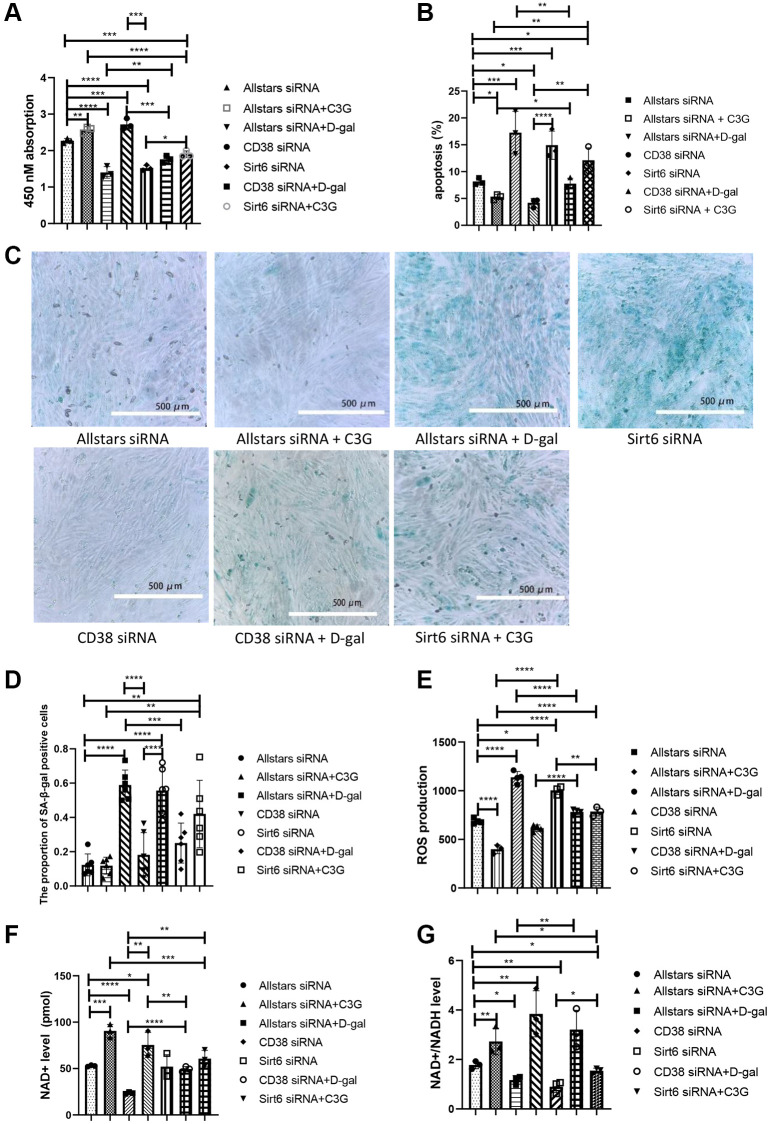
**CD38 siRNA alleviated the senescence-related index in D-gal-treated H9c2 cells, and Sirt6 siRNA aggravated the senescence-related index.** Cell proliferation was detected with the CCK-8 assay (**A**), and cell apoptosis was detected using flow cytometry (**B**). β-Gal expression was observed using cytochemistry (**C**) and semi0quantitatively analyzed (**D**). Cellular ROS levels were detected with a ROS assay (**E**), and the NAD+ level (**F**) and NAD+/NADH ratio (**G**) were detected with an NAD+/NADH detection kit. Magnification, 10×. ^*^P < 0.05, ^**^P < 0.01, ^***^P < 0.001 and ^****^P < 0.0001. We repeated this procedure 3 times.

The viability and metabolism of H9c2 cells were examined following siRNA transfection. Compared with H9c2 cells transfected with Allstars siRNA, the proliferation of cells transfected with CD38 siRNA or Allstars siRNA followed by C3G treatment was significantly increased, while cell proliferation was decreased in cells transfected with Sirt6 siRNA or Allstars siRNA followed by D-gal treatment. Compared to cells transfected with CD38 siRNA, the proliferation of cells transfected with CD38 siRNA followed by D-gal treatment was reduced. The proliferation of cells transfected with Allstars siRNA, Allstars siRNA followed by C3G treatment or Sirt6 siRNA followed by C3G treatment was significantly increased compared with that of cells transfected with Sirt6 siRNA (Figure 3A). Compared with H9c2 cells transfected with Allstars siRNA, apoptosis was significantly increased in cells transfected with Allstars siRNA followed by D-gal treatment or transfected with Sirt6 siRNA but was significantly reduced in cells transfected with CD38 siRNA or Allstars siRNA followed by C3G treatment. Apoptosis was significantly increased in cells treated with Allstars siRNA followed by D-gal compared to cells transfected with CD38 siRNA. Apoptosis was significantly decreased in cells transfected with Allstars siRNA, CD38 siRNA or Allstars siRNA followed by C3G treatment compared to cells transfected with Sirt6 siRNA. D-gal-treated cells showed reduced apoptosis following transfection with CD38 siRNA compared to cells transfected with Allstars siRNA followed by D-gal treatment. Compared with cells transfected with Allstars siRNA followed by C3G treatment, cells transfected with Sirt6 siRNA followed by C3G treatment showed significantly increased levels of apoptosis (Figure 3B). The expression levels of SA-*β*-galactosidase were examined using cytochemistry. SA-*β*-galactosidase expression was significantly higher in H9c2 cells treated with D-gal after transfection with Allstars siRNA or Sirt6 siRNA than in the cells transfected with Allstars siRNA. SA-*β*-galactosidase expression was also higher in cells treated with D-gal after transfection with Allstars siRNA or Sirt6 siRNA than in cells transfected with CD38 siRNA. SA-*β*-galactosidase expression was significantly higher in cells treated with D-gal than in cells treated with D-gal after transfection with CD38 siRNA. Allstars siRNA-transfected cells and Allstars siRNA-transfected cells treated with C3G or CD38 siRNA-transfected cells exhibited significantly lower SA-*β*-galactosidase expression than Sirt6 siRNA-transfected cells (Figure 3C and 3D). ROS levels in cells transfected with CD38 siRNA were lower than that in H9c2 cells transfected with Allstars siRNA, but were significantly higher than that in cells transfected with Allstars siRNA followed by D-gal treatment. ROS levels were higher in cells transfected with Allstars siRNA followed by D-gal treatment or CD38 siRNA followed by D-gal treatment than in cells transfected with CD38 siRNA. Cells transfected with CD38 siRNA followed by D-gal treatment showed lower ROS levels than cells transfected with Allstars siRNA followed by D-gal treatment. ROS levels were significantly lower in cells transfected with Sirt6 siRNA and subsequently treated with C3G than in cells transfected with Sirt6 siRNA. Cells transfected with Sirt6 siRNA following C3G treatment showed higher ROS levels than cells transfected with Allstars siRNA and then treated with C3G (Figure 3E). Therefore, CD38 expression reduced cell viability and promoted cell senescence and cell senescence-related metabolism, and Sirt6 expression and C3G increased cell viability and inhibited cell senescence and cell senescence-related metabolism. CD38 and C3G may exert their effects through Sirt6.

Intracellular NAD^+^ levels and NAD^+^/NADH ratios were measured in H9c2 cells. The intracellular NAD^+^ level was obviously lower in cells transfected with Allstars siRNA followed by D-gal treatment than in cells transfected with Allstars siRNA, and the NAD^+^ level was higher in cells transfected with Allstars siRNA followed by C3G treatment or CD38 siRNA transfection. NAD^+^ levels were significantly higher in cells transfected with Allstars siRNA, Allstars siRNA followed by C3G treatment, CD38 siRNA or CD38 siRNA followed by D-gal treatment than in cells transfected with Allstars siRNA following D-gal treatment. Cells transfected with CD38 siRNA followed by D-gal treatment had lower NAD+ levels than cells transfected with CD38 siRNA. Cells transfected with Sirt6 siRNA followed by C3G treatment had significantly higher NAD^+^ levels than cells transfected with Sirt6 siRNA (Figure 3F). The NAD^+^/NADH ratio was significantly lower in cells transfected with Allstars siRNA followed by D-gal treatment or Sirt6 siRNA than in H9c2 cells transfected with Allstars siRNA, while the ratio in cells transfected with Allstars siRNA followed by C3G treatment or CD38 siRNA was significantly increased. Cells transfected with Allstars siRNA followed by C3G treatment or CD38 siRNA followed by D-gal treatment displayed a significantly higher NAD^+^/NADH ratio than cells transfected with Allstars siRNA followed by D-gal treatment. The NAD+/NADH ratio of cells transfected with Sirt6 siRNA followed by C3G treatment was significantly higher than that of Sirt6 siRNA-transfected cells (Figure 3G). The results indicated that D-gal treatment and CD38 expression decreased intracellular NAD^+^ levels and the NAD^+^/NADH ratio, and Sirt6 expression and C3G increased NAD^+^ levels and the NAD^+^/NADH ratio.

### CD38 downregulates Sirt6 expression in D-gal-induced senescent cells

**Figure 4:**
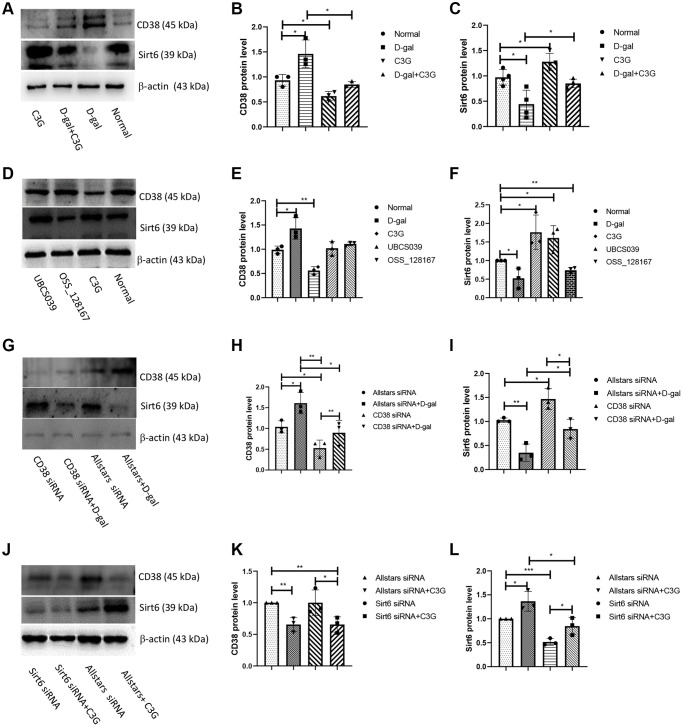
**D-gal induced CD38 expression and decreased Sirt6 expression, whereas C3G decreased CD38 expression and increased Sirt6 expression in H9c2 cells.** H9c2 cells were treated with D-gal or C3G, CD38 and Sirt6 protein expression was examined using Western blotting (**A**), and the levels of CD38 (**B**) and Sirt6 (**C**) were analyzed by normalizing β-actin expression. H9c2 cells were treated with OSS-128167 and UBCS039, the protein expression was examined (**D**), and the levels of CD38 (**E**) and Sirt6 (**F**) in the cells were analyzed. H9c2 cells were transfected with CD38 siRNA, protein expression was examined (**G**), and the levels of CD38 (**H**) and Sirt6 (**I**) in the cells were analyzed. H9c2 cells were transfected with Sirt6 siRNA, protein expression was examined (**J**), and the levels of CD38 (**K**) and Sirt6 (**L**) in the cells were analyzed. *β*-Actin was used as an internal reference. ^*^P < 0.05, ^**^P < 0.01 and ^***^P < 0.001.

Western blot analysis was performed to determine CD38 expression and Sirt6 expression in senescent H9c2 cells. Cells treated with D-gal exhibited higher levels of the CD38 protein and decreased Sirt6 protein levels than normal cells, while cells treated with C3G displayed significantly lower CD38 protein expression and higher Sirt6 expression. Cells treated with both D-gal and C3G showed a significantly lower CD38 expression level and higher Sirt6 expression than the D-gal-treated cells (Figure 4A–4C). H9c2 cells treated with UBCS039 or OSS-128167 showed no significant differences in CD38 expression levels compared to normal cells, while D-gal-treated cells showed increased CD38 expression, and C3G-treated cells showed significantly decreased CD38 expression. Sirt6 expression was significantly increased in UBCS039-treated cells and C3G-treated cells, while Sirt6 expression was decreased in D-gal-treated cells and OSS-128167-treated cells (Figure 4D–4F). Cells transfected with Allstars siRNA followed by D-gal treatment showed significantly higher CD38 protein levels than cells transfected with Allstars siRNA. Cells transfected with Allstars siRNA, CD38 siRNA or CD38 siRNA followed by D-gal treatment had lower CD38 expression levels than cells treated with Allstars siRNA and D-gal. Sirt6 protein expression was significantly increased in cells transfected with CD38 siRNA compared with cells transfected with Allstars siRNA, while the expression was significantly decreased in cells transfected with Allstars siRNA followed by D-gal treatment. Sirt6 had higher expression in cells transfected with CD38 siRNA or CD38 siRNA followed by D-gal treatment than in cells transfected with Allstars siRNA followed by D-gal treatment (Figure 4G–4I). CD38 had lower protein expression in cells transfected with Allstars siRNA followed by C3G treatment or Sirt6 siRNA followed by C3G treatment than in cells transfected with Allstars siRNA. CD38 protein expression levels were lower in cells transfected with Sirt6 siRNA followed by C3G treatment than in cells transfected with Sirt6 siRNA. Cells transfected with Sirt6 siRNA showed lower Sirt6 expression than cells transfected with Allstars siRNA. Sirt6 expression was significantly higher in cells transfected with Sirt6 siRNA followed by C3G treatment, Allstars siRNA or Allstars siRNA followed by C3G treatment than in cells transfected with Sirt6 siRNA (Figure 4J–4L). The above results indicated that CD38 expression increased and Sirt6 expression decreased in D-gal-treated senescent cells. C3G treatment decreased CD38 expression and increased Sirt6 expression. CD38 downregulated Sirt6 expression. Sirt6 activity did not change CD38 expression but changed Sirt6 expression levels.

CD38+ cells were detected using flow cytometry. The number of H9c2 cells expressing CD38 was significantly increased in cells treated with D-gal compared to normal cells, while the number of H9c2 cells expressing CD38 was significantly decreased after C3G treatment or a combination of D-gal and C3G treatment compared to D-gal treatment (Supplementary Figure 3). The results indicated that the number of CD38-expressing cells increased during senescence, and C3G treatment decreased the number of CD38-expressing cells.

CD38 and Sirt6 expression in H9c2 cells was also measured using real-time PCR. D-gal-treated cells exhibited higher CD38 mRNA expression than normal cells, while C3G-treated cells presented lower CD38 mRNA expression. CD38 mRNA levels were lower in D-gal- and C3G-treated cells or in UBCS039-treated cells than in D-gal-treated cells. CD38 mRNA expression was lower in cells treated with both OSS-128167 and C3G or C3G-treated cells than in OSS-128167-treated cells (Supplementary Figure 4A). Sirt6 mRNA expression was significantly higher in C3G-treated cells and UBCS039-treated cells than in normal cells. Sirt6 mRNA expression was higher in cells treated with both D-gal and UBCS039, C3G and D-gal-treated cells, UBCS039-treated cells and C3G-treated cells than in D-gal-treated cells. C3G-treated cells and cells treated with both OSS-128167 and C3G exhibited higher Sirt6 mRNA expression than cells treated with OSS-128167 alone. Sirt6 mRNA expression was significantly lower in normal cells than in UBCS039-treated cells (Supplementary Figure 4B). The above results correspond to the Western blotting results.

mRNA expression of Sirt1, Sirt3 and Sirt6 was also detected in H9c2 cells using real-time PCR. Sirt1, Sirt3 and Sirt6 exhibited lower mRNA expression levels in D-gal-treated cells than in normal cells, while C3G-treated cells presented increased Sirt1 and Sirt6 mRNA expression. Sirt1 and Sirt6 had higher mRNA expression in cells transfected with CD38 siRNA than in H9c2 cells transfected with Allstars siRNA (Supplementary Figure 5). The results indicated that Sirt1, Sirt3 and Sirt6 expression decreased in senescent cells. C3G treatment only increased Sirt1 and Sirt6 expression but not Sirt3 expression. CD38 siRNA downregulates Sirt1 and Sirt6 expression.

### Increased CD38 expression and decreased Sirt6 expression are involved in the aging process

**Figure 5:**
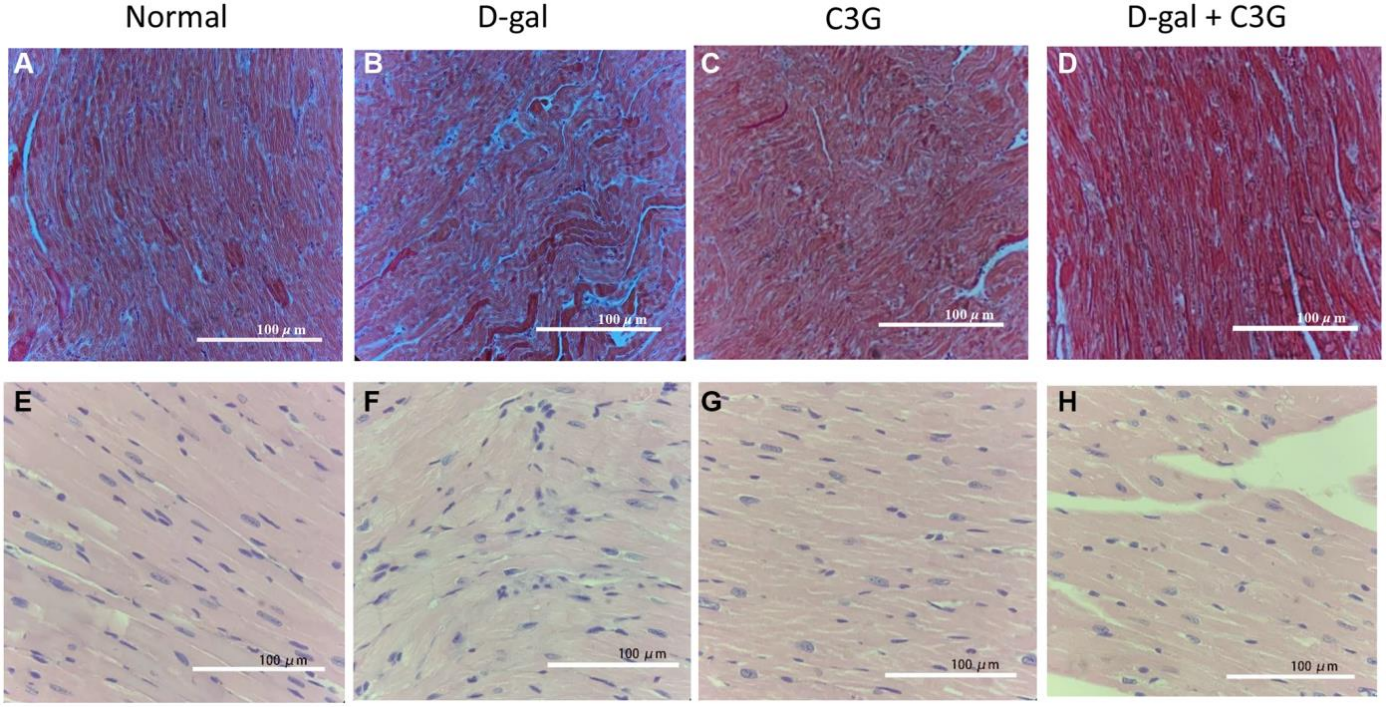
**D-gal induced aging in mouse myocardial tissues, and C3G alleviated the tissue injury.** Histological sections of cardiac tissues were stained with Masson's trichrome (**A**–**D**) or hematoxylin-eosin (**E**–**H**). Magnification, 10×.

A mouse model of D-gal-induced acute aging was established. Masson’s trichrome staining showed that the myocardium of these D-gal-injected mice exhibited significant blue collagen staining. In contrast, less blue staining was observed in myocardial tissues from C3G-treated mice and mice treated with both D-gal and C3G. HE staining showed a disordered arrangement of fibers, fractured muscle fibers and enlarged intercellular space in the myocardium of D-gal-injected mice. However, few breaks in fibers were observed in myocardial tissues of mice treated with both D-gal and C3G (Figure 5). We dissected all mice and obtained similar observations in these myocardial tissue sections. These observations indicated that D-gal successfully induces acute aging in the mouse model and that C3G exerts a therapeutic effect on aging in this animal model.

Lymphocyte subtypes and cytokines were examined in the mouse model. D-gal-treated mice showed significantly higher serum levels of IL-1α, IL-1*β*, IL-6, IL-10, IL-17A, IL-27 and TNF-α than normal mice. The mice treated with both D-gal and C3G had significantly lower levels of IL-1α, IL-1*β*, IL-6, IL-17A, TNF-α and IL-10 than the D-gal-treated mice. Additionally, the total number of white blood cells and the proportions of T cells, B cells, lymphocytes, neutrophils and mononuclear cells were not significantly different among normal mice, D-gal-treated mice, C3G-treated mice and mice treated with both D-gal and C3G. However, the proportion of NK cells in D-gal-treated mice was significantly lower than that in normal mice, and the proportion of NK cells in mice treated with both D-gal and C3G was significantly higher than that in D-gal-treated mice (Supplementary Figure 6). Thus, D-gal treatment stimulated inflammation-related cytokine production, and C3G restored the cytokine levels and NK-cell proportion.

The mouse model was examined by performing biochemical tests. Compared with normal healthy mice, ALT, CK and LDH levels were increased in the peripheral blood of the mice treated with D-gal. ALT, CK and LDH levels were significantly decreased in mice treated with both D-gal and C3G compared with D-gal-treated mice (Supplementary Figure 7). Additionally, the energy consumption level, entropy of respiration (RQ), O_2_ consumption and CO_2_ production of mice treated with D-gal were significantly lower than those of the mice in the other three groups. These metabolic indexes returned to normal levels in mice treated with both D-gal and C3G (Supplementary Figure 8). Based on these results, D-gal aggravated aging-related metabolism in the animals, and C3G treatment restored the metabolic condition.

**Figure 6:**
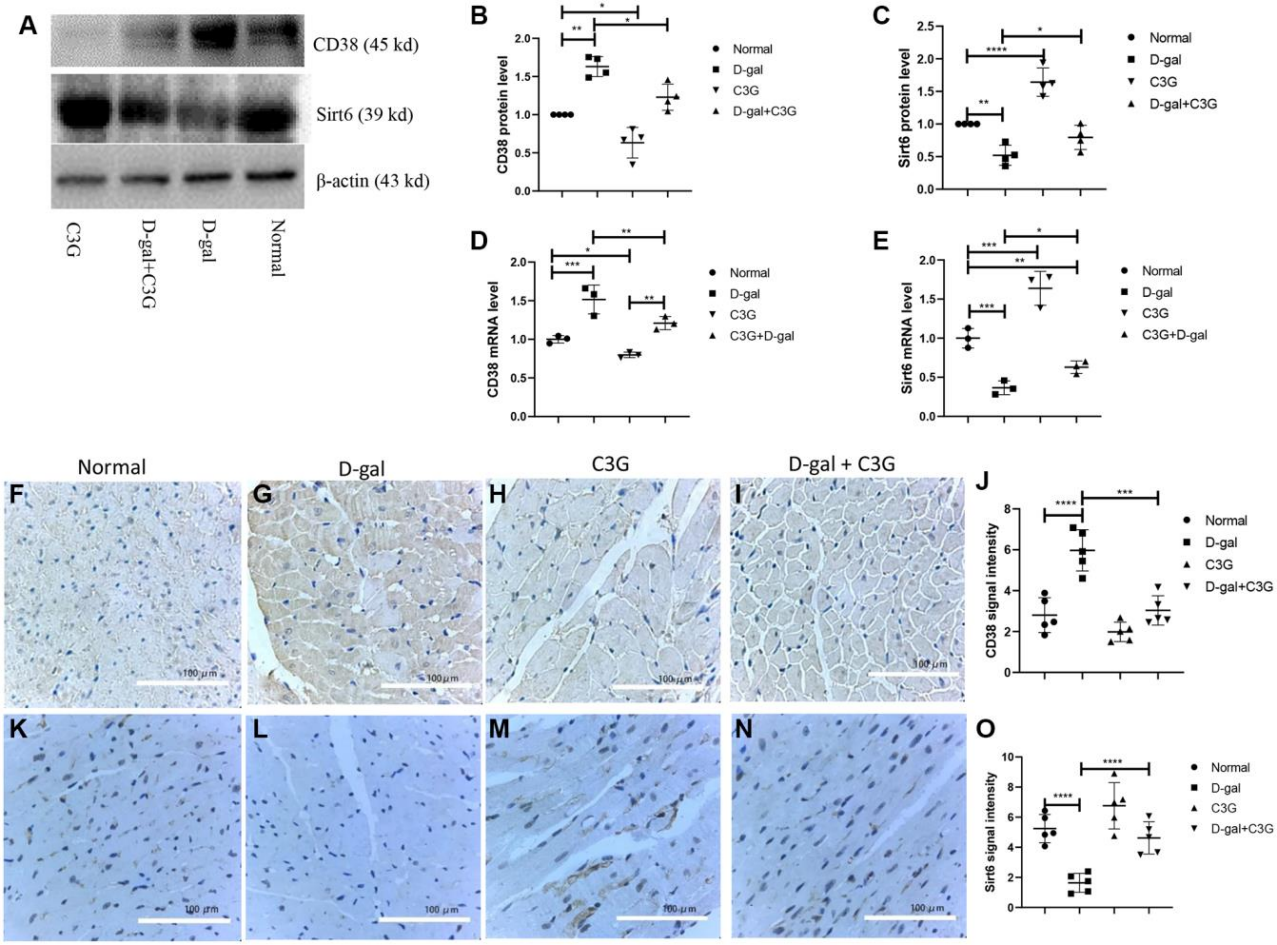
**D-gal increased CD38 expression and decreased Sirt6 expression in myocardial tissues of aging mice, and C3G decreased CD38 expression and increased Sirt6 expression in the tissues.** Protein expression was determined using Western blotting (**A**), and the levels of CD38 (**B**) and Sirt6 expression (**C**) were semi-quantitatively analyzed. Levels of the CD38 (**D**) and Sirt6 (**E**) mRNAs in mouse heart tissues were detected with real-time PCR. Immunohistochemistry was also used to detect protein expression in mouse myocardial tissues. The expression levels were semi-quantified based on immune signal density and extension. (**F**–**I**) show CD38 protein expression, and the expression was semi-quantified (**J**). (**K**–**N**) show Sirt6 protein expression, and the expression was semi-quantified (**O**). Magnification, 10 ×. ^*^P < 0.05, ^**^P < 0.01 and ^***^P < 0.001.

CD38 expression and Sirt6 expression were examined in mouse myocardial tissues using Western blot analysis and real-time PCR. CD38 protein (45 kDa) and mRNA expression levels were higher and Sirt6 protein and mRNA expression levels were lower in myocardial tissues from D-gal-treated mice than in normal mice (Figure 6A–6E). CD38 expression and Sirt6 expression in mouse myocardial tissues were also detected using immunohistochemistry, and the signal density was semi-quantified. CD38 immunosignal staining was significantly higher in myocardial tissues from D-gal-treated mice than in normal mice, but Sirt6 staining was very low. Compared to D-gal-treated mice, the mice treated with both D-gal and C3G exhibited lower CD38 immunostaining in myocardial tissues, but the Sirt6 immunostaining signal was higher (Figure 6F–6O). Thus, D-gal treatment increased CD38 expression and decreased Sirt6 expression in mouse myocardial tissues, and C3G decreased CD38 expression and increased Sirt6 expression in aging mice.

Intracellular NAD^+^ levels and NAD^+^/NADH ratios were measured in mouse myocardial tissues. NAD^+^ levels and NAD^+^/NADH ratios in myocardial tissues of D-gal-treated mice were significantly lower than those in normal mice. The level and ratio were restored to normal levels when the mice were treated following C3G treatment, and the level and ratio were higher than those in D-gal-treated mice (Supplementary Figure 7G, 7H). This result indicated that D-gal treatment reduced NAD^+^ levels and the NAD^+^/NADH ratio in aging mice and that C3G treatment restored NAD^+^ concentrations and NAD^+^/NADH ratios to normal levels.

## DISCUSSION

Many studies have used cell proliferation, glucose consumption, ROS, TERT and SA-*β*-gal as biological markers to examine cell senescence. Abnormal cell viability is the main characteristics of senescent cells [29–32]. In the present study, D-gal significantly inhibited H9c2 cell proliferation and promoted apoptosis. OSS-128167 inhibited H9c2 cell proliferation and stimulated apoptosis, but C3G and UBCS039 reversed the D-gal- or OSS-128167-mediated inhibition of cell proliferation and induction of apoptosis. Others have also detected increased apoptosis in aging mouse model and in D-gal-induced senescent H9c2 cells [33, 34], although senescent cells are known to resist apoptosis. These results and results by others suggested that with D-gal treatment, the number of H9c2 cells decrease due to decreased cell proliferation, increased apoptosis and even necrotic cell death. The ROS and *β*-galactosidase levels are increased in cellular senescence [35–37]. TERT expression is among the most important indicators of senescent cells, and the TERT level decreases during cell senescence [38–41]. Aging is characterized by a dysregulated metabolism, which occurs with glycolysis [42–44]. Hexokinase 2 (HK2) expression in glycolysis increases during cellular senescence [45]. Thus, in some studies, HK2 was considered a senescence-related gene. Sirt6 is also involved in glycolytic metabolism [46]. The present study showed that D-gal reduced TERT expression and increased the levels of ROS, SA-*β*-galactosidase and HK2. In contrast, C3G reduced HK2, SA-*β*-galactosidase and ROS levels and increased TERT expression. OSS-128167 reduced TERT expression and increased ROS, SA-*β*-galactosidase and HK2 levels, whereas UBCS039 exerted the opposite effects in H9c2 cells. Additionally, D-gal treatment injured the structure of myocardial tissue in mice, while C3G alleviated D-gal-induced cardiac aging and restored the tissue structure in mice. Both cardiomyocyte death and senescence may be the primary injury induced by D-gal because, as observed in the mouse model in the present study, the levels of circulating CK and LDH were elevated and levels of NAD^+^ that suppressed cell death were declined. Another report found that CD38 knockdown significantly protected cardiomyocytes from D-gal-induced cellular senescence. Other group also found that OSS-128167 significantly increased the expression of collagen I, a pro-fibrotic marker, in H9c2 cells [47], which support our result. The above observations indicated that D-gal treatment promoted cellular senescence and this effect was closely related to CD38 and Sirt6 activities. Furthermore, C3G, a natural inhibitor of CD38, exerted an antiaging effect by mediating Sirt6 activity.

In D-gal-induced cell senescence, CD38 expression was increased, while Sirt6 expression was decreased. Flow cytometry also detected increased numbers of CD38-positive H9c2 cells following the D-gal treatment. Importantly, transfection of CD38 siRNA increased Sirt6 expression and alleviated the inhibitory effect of D-gal on Sirt6 expression. C3G not only stimulated Sirt6 expression but also relieved the Sirt6 siRNA-mediated inhibition of Sirt6 expression in H9c2 cells. OSS-128167 inhibited Sirt6 expression, while UBCS039 increased Sirt6 expression, indicating that increased Sirt6 activity could upregulate Sirt6 expression. However, Sirt6 siRNA, OSS-128167 and UBCS039 did not alter CD38 expression. Additionally, our studies revealed that CD38 siRNA increased H9c2 cell proliferation and TERT expression and decreased apoptosis and HK2, SA-*β*-galactosidase and ROS levels. Sirt6 siRNA exerted the opposite effects. C3G alleviated the inhibitory effect of Sirt6 siRNA on TERT expression and the stimulatory effect on SA-*β*-galactosidase expression. In D-gal-induced aging mice, CD38 expression was increased in myocardial tissues, while the Sirt6 expression was decreased. C3G treatment reduced CD38 expression and increased Sirt6 expression in the heart tissue. These *in vitro* and *in vivo* results suggested that D-gal promoted cellular senescence by upregulating CD38 expression and downregulating Sirt6 expression. C3G plays an antiaging role by inhibiting CD38 expression and subsequently increasing Sirt6 expression. Additionally, we detected high levels of NAD+ and NAD^+^/NADH in H9c2 cells treated with CD38 siRNA or C3G and low levels of NAD+ in the cells treated with D-gal. We also detected decreased levels of NAD+ and NAD/NADH in D-gal-induced aging mice and increased levels of NAD^+^ and NAD^+^/NADH in the mouse model following C3G treatment. Thus, we suggest that CD38 decreased NAD+ levels and Sirt6 expression to stimulate cell senescence. Other researchers also detected the expression of CD38 in H9C2 cells [48]. Some studies reported that FoxO3a, a transcription factor, bound to the promoter region of Sirt6 and enhanced its expression [49, 50]. Functional studies revealed that Akt inactivation increased FoxO3a activity to bind Sirt6 promoter, leading to increased expression of Sirt6 [50]. Interestingly, CD38 activates the PI3K/AKT/mTOR signaling in cervical cancer cells [51]. Thus, it is possible that CD38 down-regulates FoxO3a by Akt signaling to suppress Sirt6 expression.

The senescence-associated secretary phenotype (SASP) is the key feature of aging cells and includes a series of proinflammatory cytokines, chemokines and proteases [52–54]. Senescent cells secrete a series of inflammatory cytokines, chemokines, growth factors and matrix remodeling factors that alter local tissue environments [52–55]. In our study, serum IL-α, IL-1*β*, IL-6, IL-10, IL-17A, IL-27 and TNF-α levels, as well as ALT, CK and LDH levels, were significantly increased in D-gal-treated mice. After C3G treatment, the levels of these cytokines and metabolites were significantly decreased. Interestingly, a decrease in the NK-cell proportion was detected in the peripheral blood of mice following D-gal treatment, and C3G treatment restored the proportion of NK cells to a normal level. These results supported that the alternative production of these proinflammatory cytokines and metabolism-related enzymes was closely related to aging. Proinflammatory cytokines induce inflammation to accelerate aging [52–54]. The above results also indicate that D-gal contributed to aging-related immunity and metabolism, and C3G treatment restored the immune and metabolic conditions. Chronic inflammation is a crucial driver for SASP, which accumulates during aging and in age-related diseases. SASP mediates chronic inflammation, which causes damage to tissue structures [56]. Cytokine dysregulation may play a key role in remodeling the immune system at older ages [57]. Proinflammatory cytokines, such as TNF-α, amplifies senescence-associated inflammation and induce senescence [58]. Additionally, metabolism plays an essential role in cell senescence, and alternative metabolism is an important part of cell senescence [55, 59]. The metabolic alterations of glucose, lipid, glutamine and NAD+ can facilitate or inhibit cell senescence [9]. Sirtuins play versatile roles in metabolic disorders from the modulation of mitochondrial function to therapeutic interventions [60]. Sirt6 serves as a polyhedron in glycolytic metabolism and aging-related diseases [46]. Thus, as a CD38 inhibitor, C3G could alter metabolism and suppress proinflammation via the CD38-Sirt6 pathway, which ultimately contributes to SASP and cell senescence.

The Sirt family plays important roles in cell senescence [61]. Other studies detected decreased Sirt1 expression in heart tissues from old mice. Inhibiting Sirt1 expression partially reversed the effects of CD38 knockdown on D-gal-induced senescence [62, 63]. Our study also detected increased expression of Sirt1 and Sirt6 but not Sirt3 in H9c2 cells in the presence of CD38 siRNA and C3G. These results suggested that CD38 may modulate both Sirt1 and Sirt6 expression, although we cannot determined which signaling pathway is more important for cellular senescence. Recent studies highlighted the pleiotropic protective actions of Sirt6 in angiogenesis and cardiovascular diseases, including atherosclerosis, hypertension, heart failure and stroke. Mechanistically, Sirt6 participates in vascular diseases via epigenetic regulation of endothelial cells, vascular smooth muscle cells and immune cells [64]. Sirt6 has emerged as a promising target for the development of small-molecule activators and inhibitors possessing therapeutic potential in diseases ranging from cancer to age-related disorders [65–67]. Our study provides a novel mechanism involving the CD38-Sirt6 pathways in aging.

CD38 expression also effects abnormalities in the immune system that are related to aging. We previously investigated the regulatory mechanism of CD38 in NK cells and the corresponding effect on Treg cell differentiation in a rat model with collagen-induced arthritis, which is an animal model for rheumatoid arthritis and autoimmune diseases; this was performed because immune balance and imbalance are closely related to aging. In CD38-expressing NK cells, C3G treatment significantly decreased the expression of CD38 and production of IFN-γ, while the expression of Sirt6 and level of tumor necrosis factor (TNF)-α were increased; this result is similar to that observed for H9c2 cells. When CD38+ NK cells were treated with Sirt6 siRNA, the level of TNF-α sharply decreased and the level of IFN-γ increased. This result confirmed that CD38 regulated the production of inflammatory cytokine by regulating the expression of Sirt6 in NK cells. Furthermore, we found that NK cells expressing CD38 significantly inhibited CD4+ T cell differentiation to Treg cell proportion and IL-10 production. With a C3G pretreatment, CD38+ NK cells lost their ability to inhibit Treg differentiation from CD4+T cells. In rats with collagen-induced arthritis, CD38+ NK cell proportion was significantly increased and Treg cell proportion was decreased. When the animal was treated with C3G, Treg cell proportion was elevated, joint inflammation was significantly alleviated and the immune balance, including the Th1/Th2 and Th17/Treg ratio, was restored. When CIA rats were injected with both C3G and the Sirt6 inhibitor OSS_128167, the rats exhibited joint inflammation and a low Treg cell proportion, indicating that CD38 effect immune balance by mediating Sirt6 activity in the animal model [15, 68]. Increasing evidence shows that Treg cells are deeply involved in aging and various age-related diseases [69]. Treg cells’ dysfunction occurs in patients with advanced ages [70]. Additionally, autoimmunity increases with age. At older ages, the immune system cannot adequately control autoimmunity [71]. Our results suggest that CD38-Sirt6 pathway not only plays important role in aging-related heart diseases, but is also involved in abnormal immunity in many aging-related diseases.

The compound OSS-128167 is a specific Sirt6 inhibitor [24]. OSS-128167 is a selective compound for Sirt6, as its IC50 values for Sirt6 were approximately 20-fold higher than that for Sirt1 and Sirt2. UBCS039 is the first synthetic, specific Sirt6 activator. Both OSS-128167 and UBCS039 affect the acetylation of Sirt6-targeted proteins by inhibiting or stimulating Sirt6 activity. For example, UBCS039 induces deacetylation of Sirt6-targeted histone H3 sites in human cancer cells [26]. In our study, we treated H9c2 cells with Sirt6 activator UBS039, Sirt6 inhibitor OSS-128167 and Sirt6 siRNA to confirm the specificity of the CD38-Sirt6 pathway in cellular senescence and to prevent these chemicals from potentially cross reacting with other targets. Additionally, we used different concentrations of D-gal to treat the mouse model and found that 100 mg/kg D-gal showed the best senescence-inducing effect. Many studies also used this a concentration of D-gal to induce acute aging in mouse model. In the most recently published articles, mouse models were treated with even higher concentrations [72, 73].

## CONCLUSIONS

D-gal increases CD38 expression in myocardial cells and tissues, which inhibits Sirt6 expression to induce cellular senescence. C3G exerts an antiaging effect by mediating the CD38-Sirt6 pathway.

## Supplementary Materials

Supplementary Figures

Supplementary Table 1
