# Supplementary material for: Downregulation of Sirt6 by CD38 promotes cell senescence and aging
Source: Aging (Albany NY). 2022 Dec 6;14(23):9730–57. doi: 10.18632/aging.204425 (PMC9792202; doi:10.18632/aging.204425)
Supplement: Supplementary Figures [file aging-14-204425-s001.pdf]

SUPPLEMENTARY FIGURES

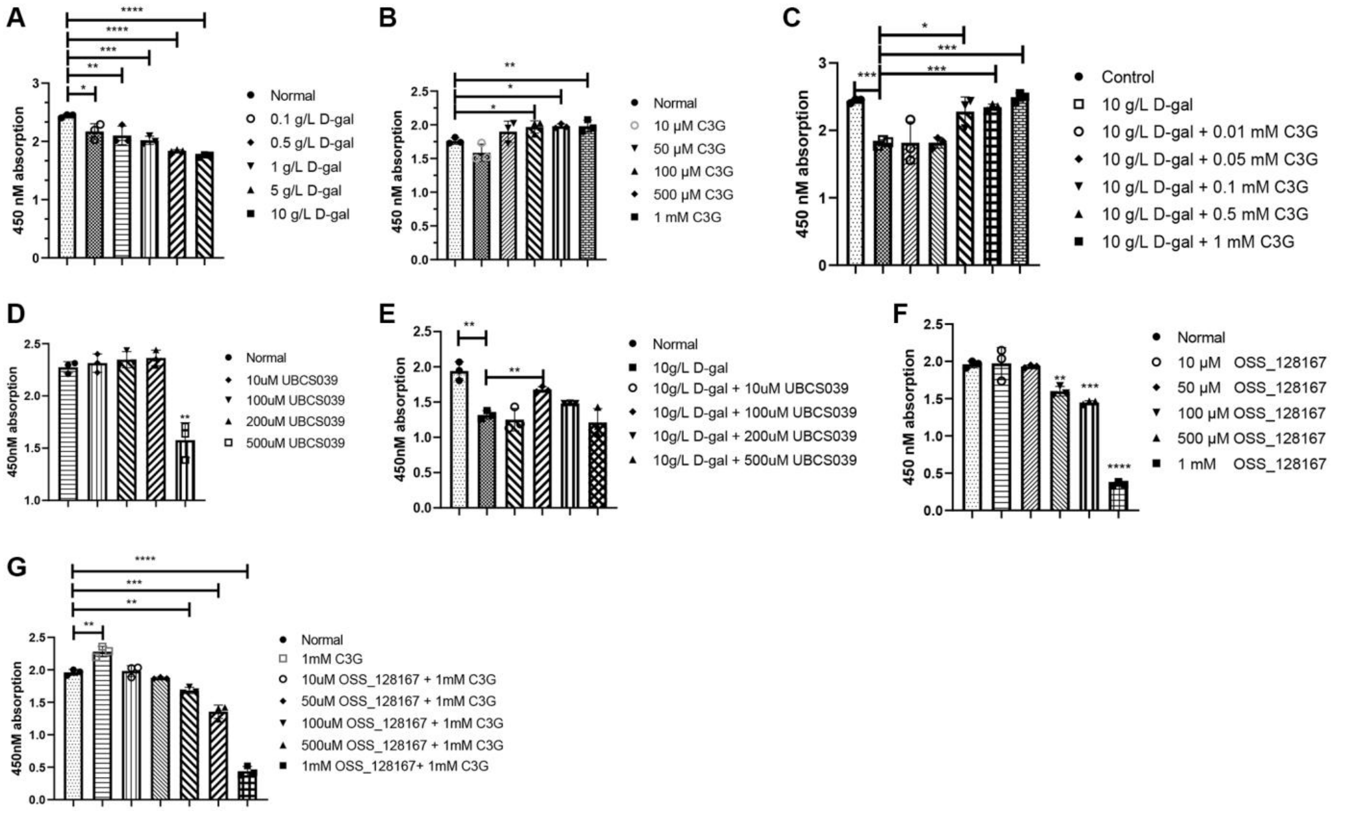

Supplementary Figure 1. D-gal inhibited H2c2 cell proliferation, and C3G increased cell proliferation, as shown by a CCK-8 assay. (**A**) The effects of D-gal at different concentrations on cell proliferation. (**B**) The effects of C3G at different concentrations on cell proliferation. (**C**) The effects of D-gal in combination with C3G on cell proliferation. (**D**) The effects of UBCS039 at different concentrations on cell proliferation. (**E**) The effects of UBCS039 in combination with D-gal on cell proliferation. (**F**) The effects of OSS_128167 at different concentrations on cell proliferation. (**G**) The effects of OSS_128167 in combination with C3G on cell proliferation $^{*}P < 0.05$, $^{**}P < 0.01$, $^{***}P < 0.001$ and $^{****}P < 0.0001$.

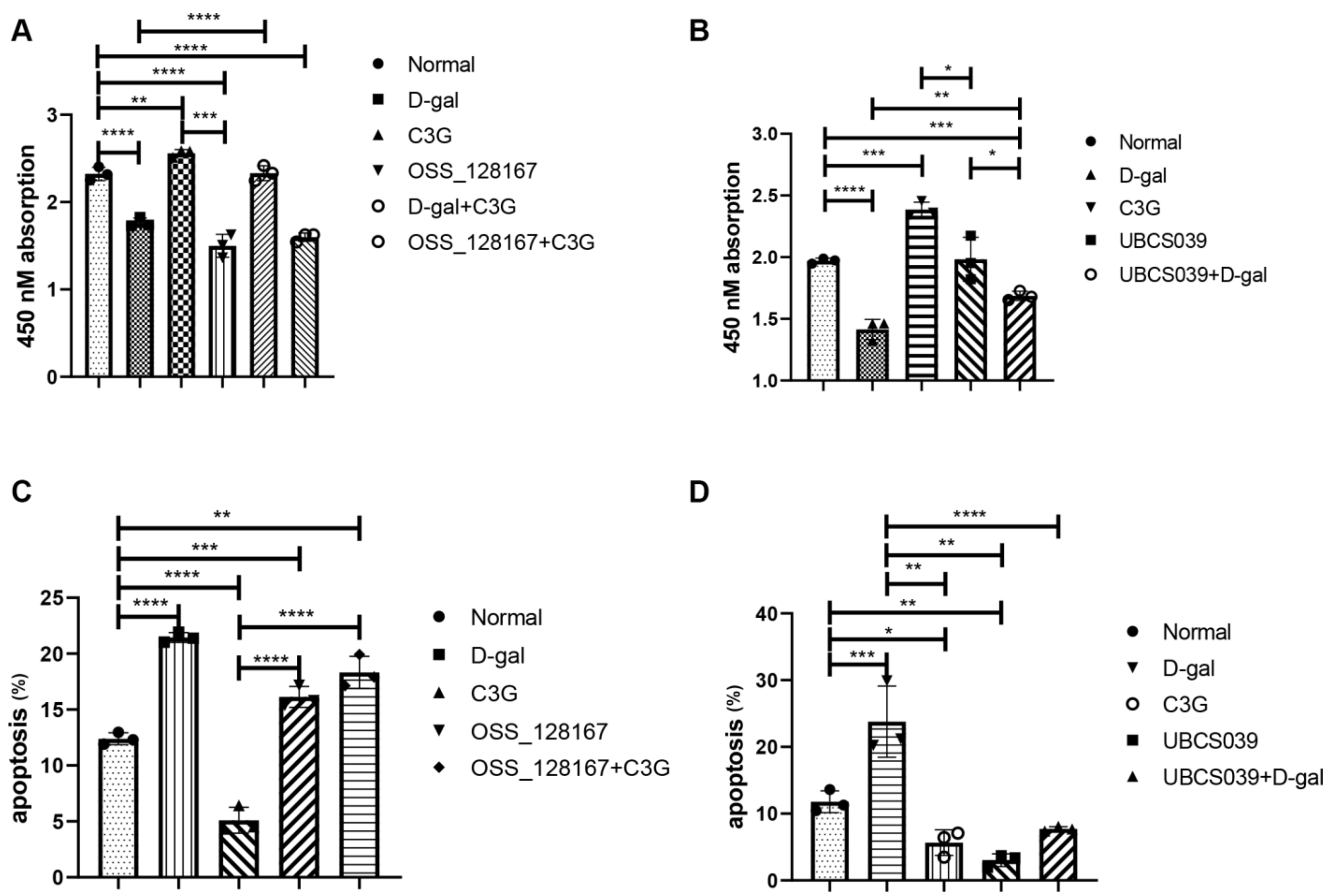

Supplementary Figure 2. D-gal and OSS-128167 increased apoptosis and decreased proliferation, and C3G and UBCS039 decreased apoptosis and increased proliferation in H9c2 cells. The effects of D-gal, OSS-128167 and C3G (**A**) and D-gal, UBCS039 and C3G (**B**) on H9c2 cell proliferation were detected with a CCK-8 assay. The effects of D-gal, OSS-128167 and C3G (**C**) and D-gal, UBCS039 and C3G (**D**) on H9c2 cell apoptosis were detected using flow cytometry. $^{*}P < 0.05$, $^{**}P < 0.01$, $^{***}P < 0.001$ and $^{****}P < 0.0001$.

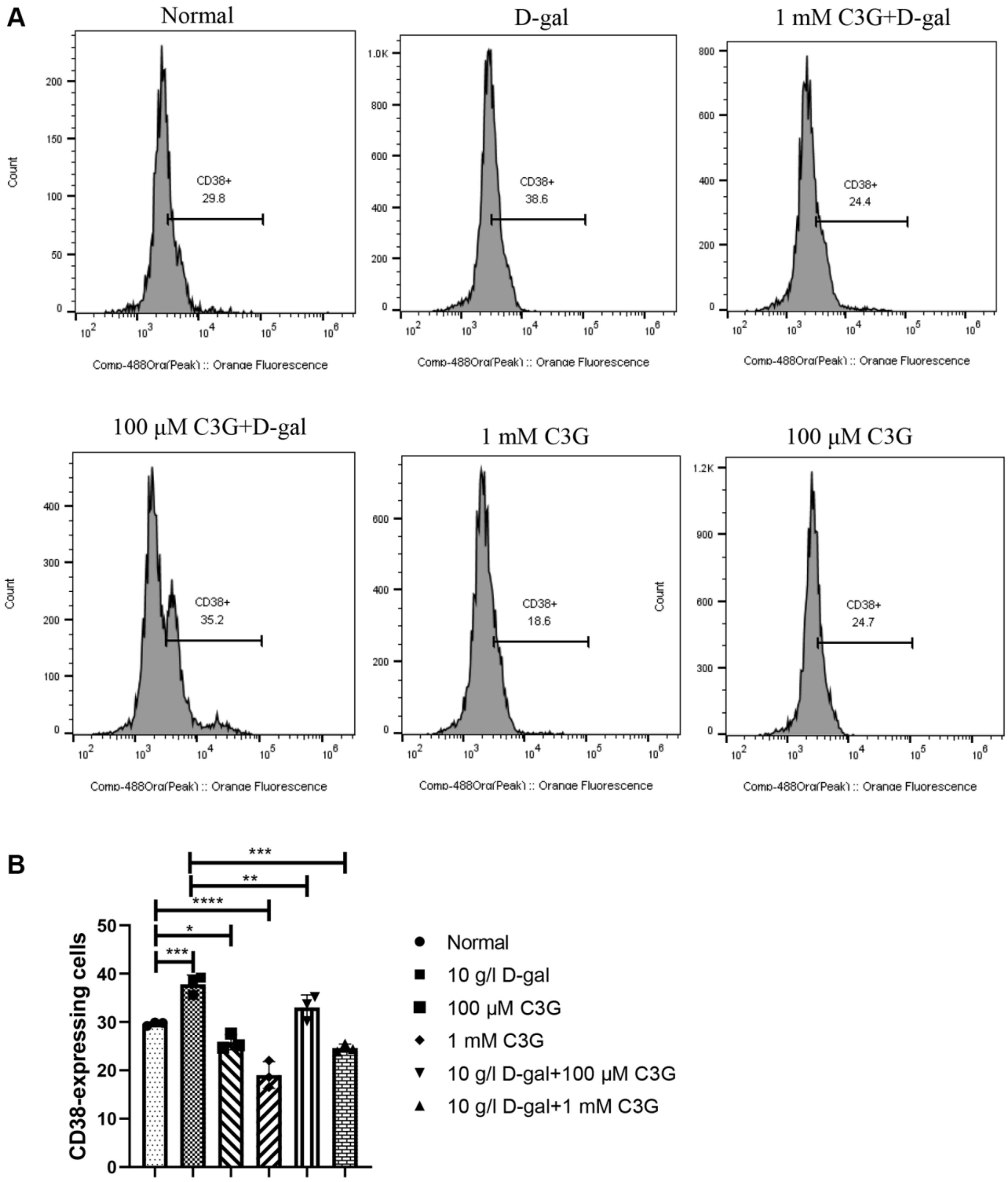

Supplementary Figure 3. D-gal increased the proportion of CD38-expressing H9c2 cells, and C3G decreased the proportion. H9c2 cells expressing CD38 were detected using flow cytometry (**A**), and the proportion of H9c2 cells expressing CD38 was quantitatively analyzed (**B**). $^{*}P < 0.05$, $^{**}P < 0.01$, $^{***}P < 0.001$ and $^{****}P < 0.0001$.

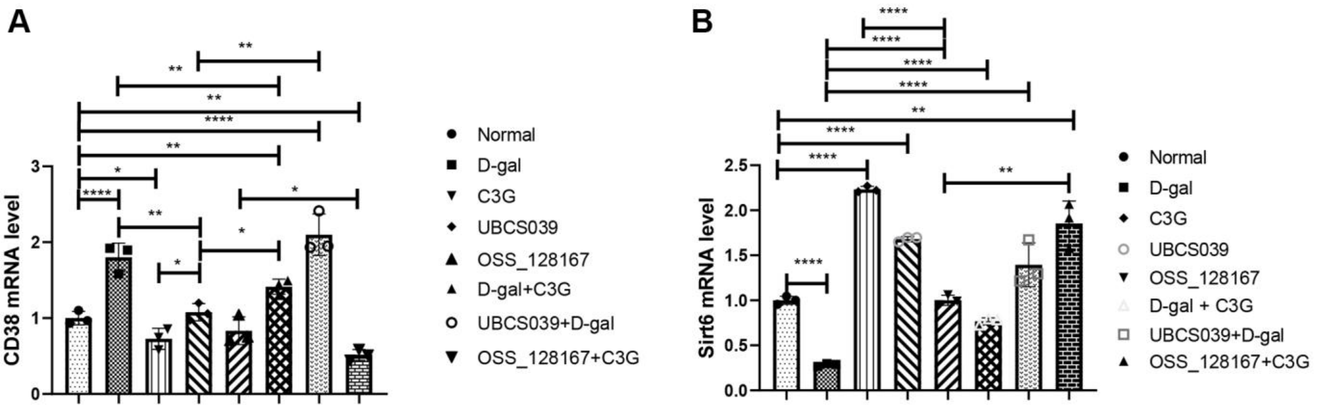

Supplementary Figure 4. CD38 mRNA expression was increased by D-gal and decreased by C3G, and Sir6 mRNA expression was decreased by D-gal and increased by C3G and UBCS039 in H9c2 cells. CD38 (**A**) and Sirt6 (**B**) mRNA expression levels were determined using real-time PCR. $^{*}P < 0.05$, $^{**}P < 0.01$, $^{***}P < 0.001$ and $^{****}P < 0.0001$.

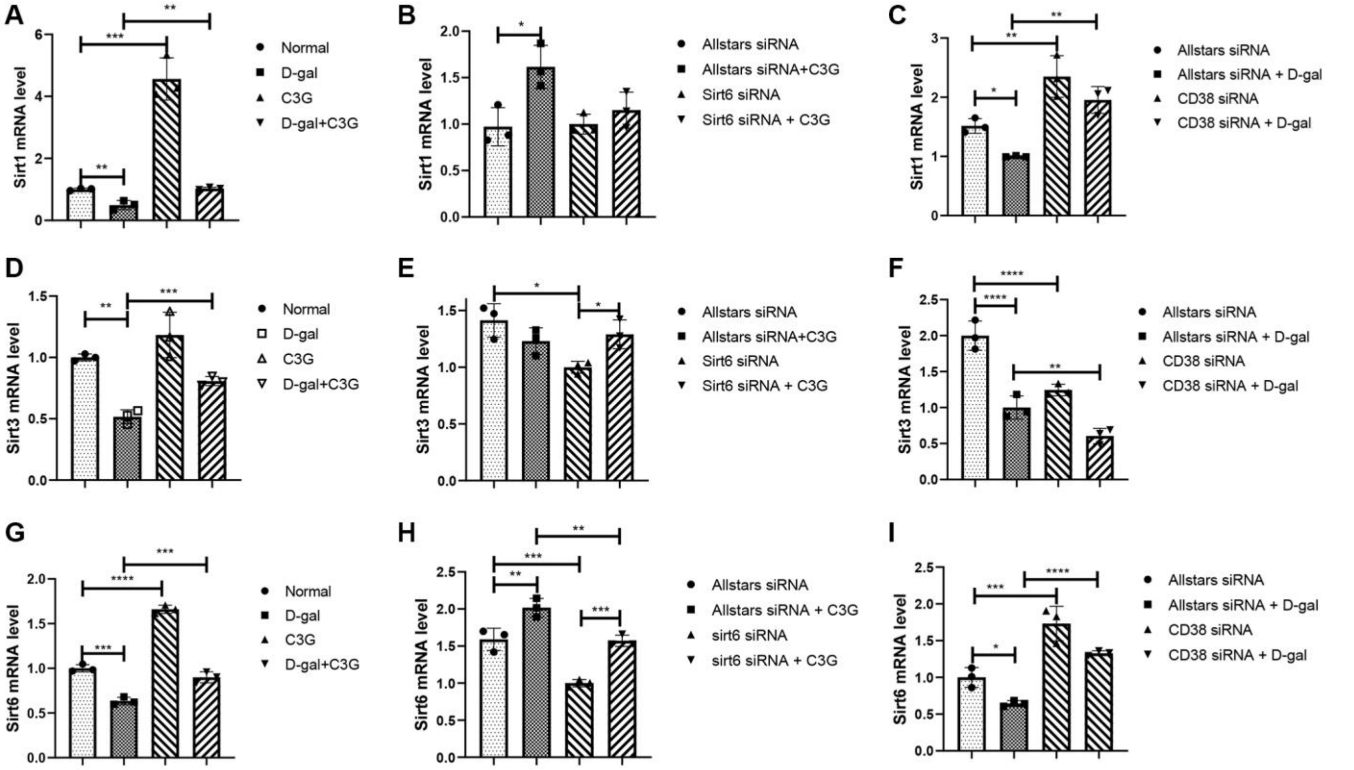

Supplementary Figure 5. The mRNA expression of Sirt1 and Sirt6 was increased in H9c2 cells in the presence of CD38 siRNA and C3G. The mRNA expression levels of Sirt1 (**A**–**C**), Sirt3 (**D**–**F**) and Sirt6 (**G**–**I**) were measured using real-time PCR. $^{*}P < 0.05$, $^{**}P < 0.01$ and $^{***}P < 0.001$.

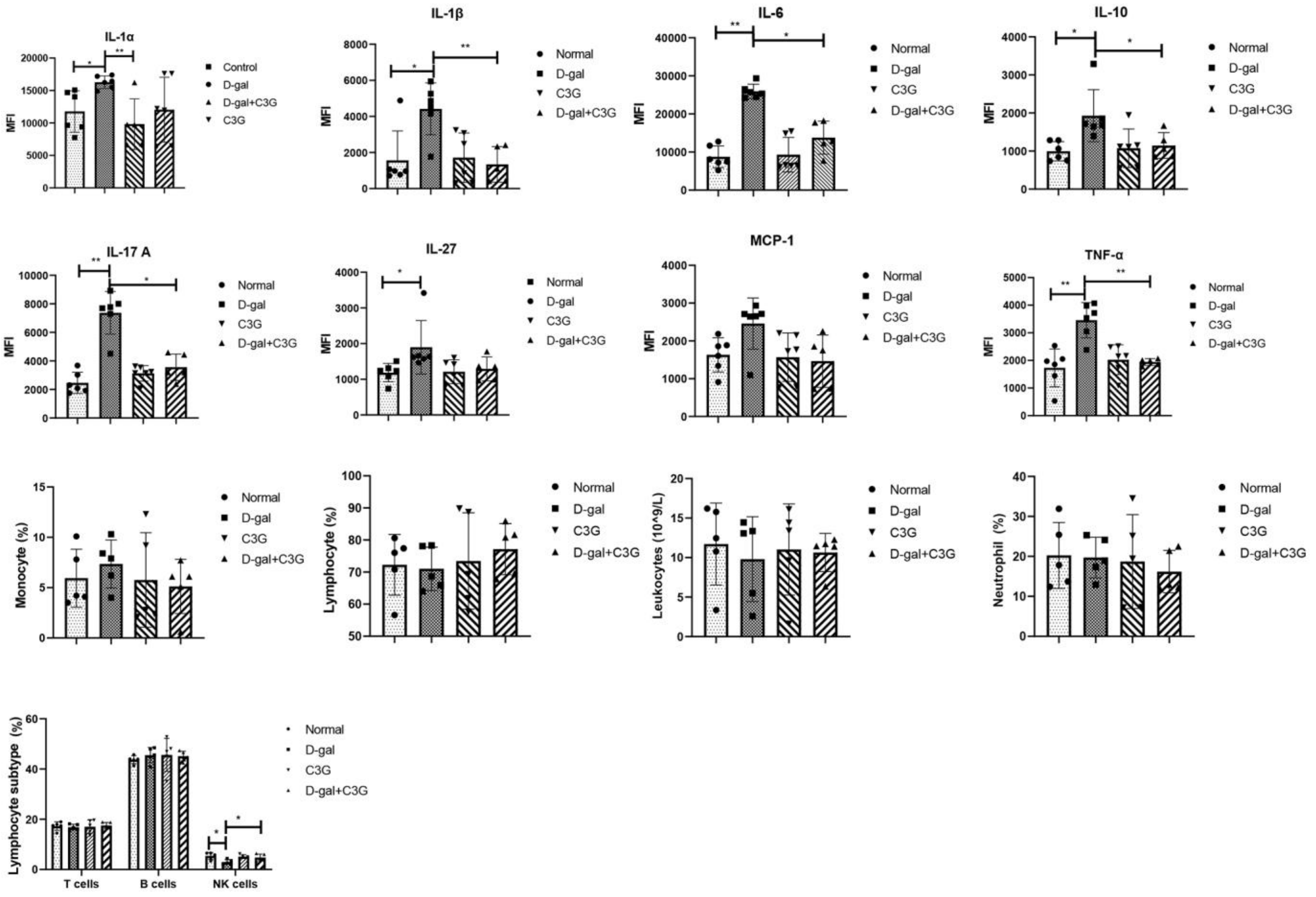

Supplementary Figure 6. D-gal and C3G altered the immunity of the treated mice. The cytokine concentrations and lymphocyte proportions in the peripheral blood of treated mice were measured using flow cytometry, and the leukocyte proportion was measured with a routine blood assay. Abbreviation: MFI: mean fluorescence intensity. $^{*}P < 0.05$, $^{**}P < 0.01$ and $^{***}P < 0.001$.

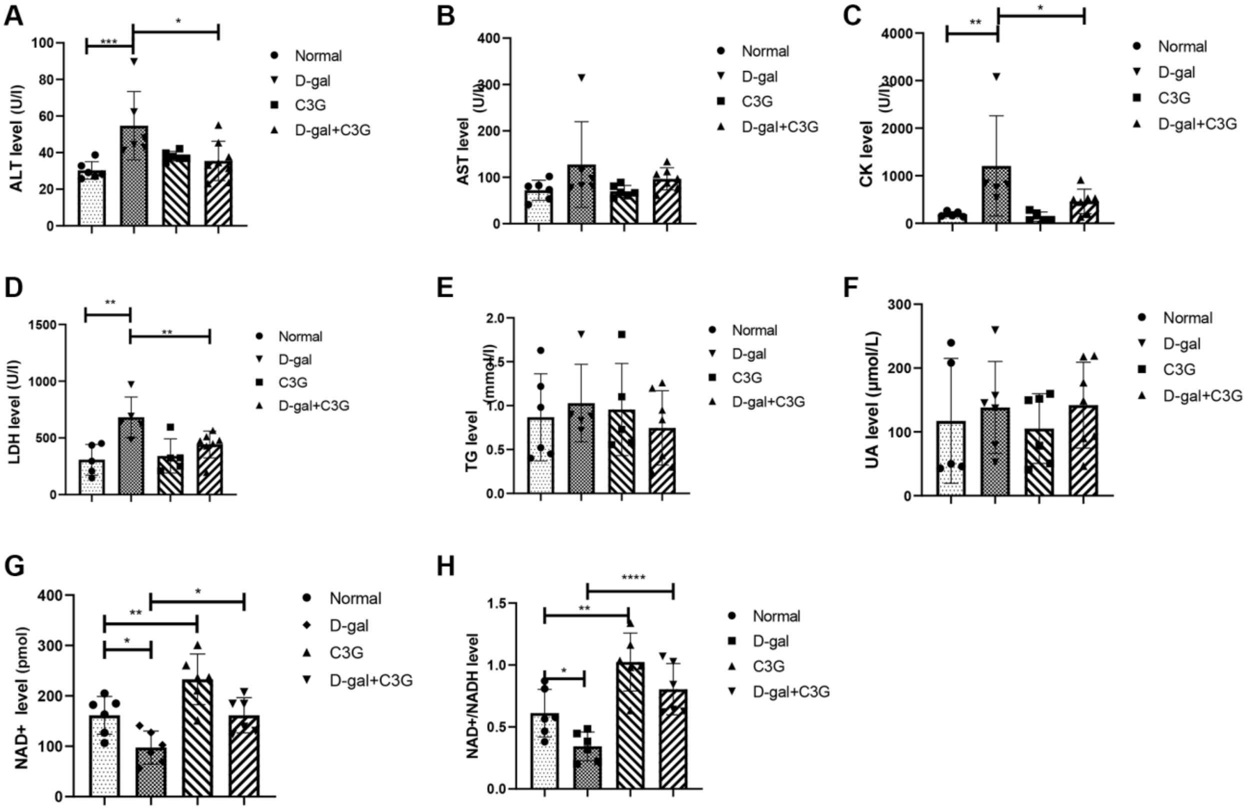

Supplementary Figure 7. D-gal and C3G treatment altered biochemical parameters in mouse peripheral blood. D-gal treatment decreased NAD+ levels in mouse myocardial tissues, and C3G treatment increased NAD+ levels. The biochemical indexes in the serum, including (**A**) alanine aminotransferase (ALT), (**B**) aspartate aminotransferase (AST), (**C**) creatine kinase (CK), (**D**) lactate dehydrogenase (LDH), (**E**) triglyceride (TG), and (**F**) uric acid (UA) levels, were measured using an automatic biochemical analyzer. (**G**) Intracellular NAD+ levels and (**H**) NAD+/NADH ratios in mouse myocardial tissues were measured using an NAD+/NADH assay kit. $^{*}P < 0.05$, $^{**}P < 0.01$ and $^{***}P < 0.001$. alanine aminotransferase (ALT), aspartate aminotransferase (AST), triglyceride (TG), uric acid (UA), and creatine kinase (CK).

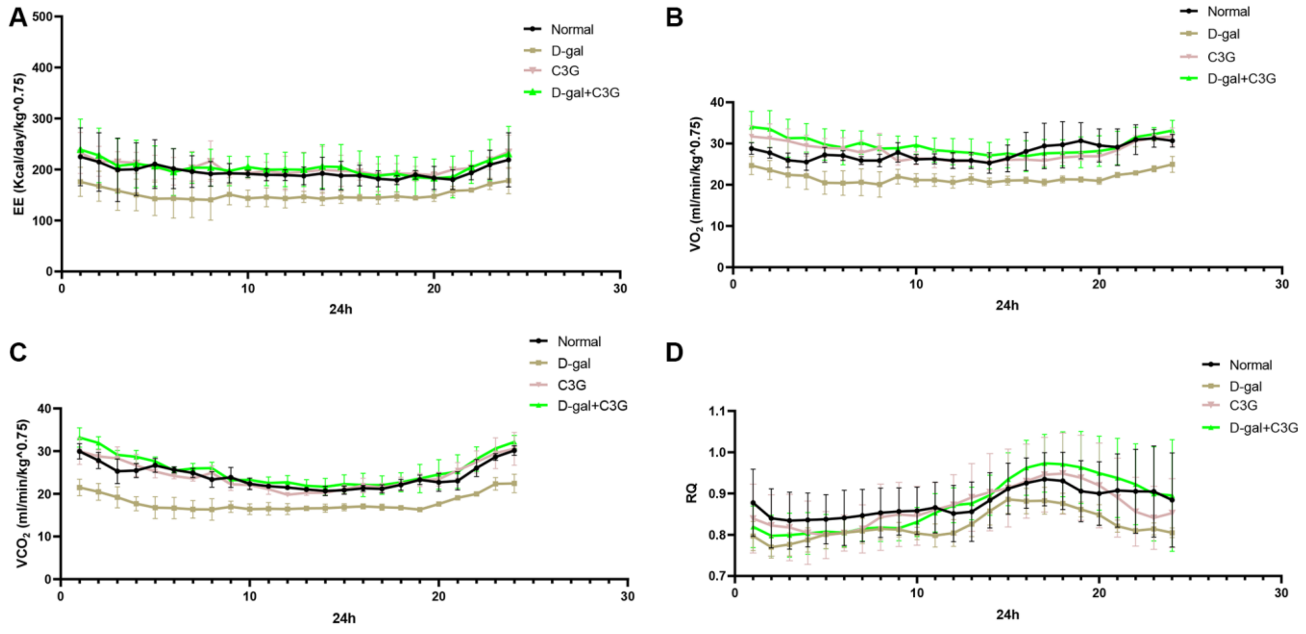

Supplementary Figure 8. D-gal treatment decreased mouse energy consumption, and C3G restored the consumption level. The levels of total energy metabolism (**A**), CO_2 production (**B**), O_2 consumption (**C**) and respiratory quotient (RQ) (**D**) were examined using an Oxylet Pro animal metabolic system.
