# Supplementary material for: Downregulation of Sirt6 by CD38 promotes cell senescence and aging
Source: Aging (Albany NY). 2022 Dec 6;14(23):9730–57. doi: 10.18632/aging.204425 (PMC9792202; doi:10.18632/aging.204425)
Supplement: Supplementary Table 1 [file aging-14-204425-s002.pdf]

SUPPLEMENTARY TABLE

Supplementary Table 1. Primer sequences for target genes.

Target gene	Sequence of primer
SIRT1 Rat	F: 5′-TGACCTCCTCATTGTTATTGGG-3′<br>R: 5′-GGCATACTCGCCACCTAACCT-3′
SIRT3 Rat	F: 5′-TCTGGTATCCCTGCCTCAAAG<br>R: 5′-CACGTCAGCCCGTATGTCTTC
SIRT6 Rat	F: 5′-TGGGTCGAATATCTCGGGCA-3′<br>R: 5′-CAGTGTTGGTGATGTCGGTG-3′
CD38 Rat	F: 5′-TGGAGCAAGTCCAAACACCTGGC-3′<br>R: 5′-CTGGGGTCTCCACACCACCTGA-3′
β-actin Rat	F: 5′-CCCATCTATGAGGGTTACGC-3′<br>R: 5′-TTTAATGTCACGCACGATTTC-3′
HK2 Rat	F: 5′-AGACCAGAGCATCCTCCTCAAGTG -3′<br>R: 5′-TCATTCACCACGGCAACCACATC-3′
TERT Rat	F: 5′-TCCTCTGTGCCCGCTGGTTAC-3′<br>R: 5′-AGCCTCTTCGTACCTCGTGATGG-3′
SIRT6 Mouse	F: 5′-GGCTACGTGGATGAGGTGAT-3′<br>R: 5′-GGCTCAGCCTTGAGTGCTAC-3′
CD38 Mouse	F:5′-GCCATTTTACAAAAACAGCACC-3′<br>R:5′-GCACAATCATCTTCAGCTCATT-3′
β-actin Mouse	F: 5′-TATCGCTGCGCTGGTCGG-3′<br>R: 5′-CCCACGATGGAGGGGAATAC-3′

Abbreviations: F: forward primer; R: reverse primer.
